# Simultaneous engineering of an enzyme's entrance tunnel and active site: the case of monoamine oxidase MAO-N†
†Electronic supplementary information (ESI) available. See DOI: 10.1039/c6sc05381e


**DOI:** 10.1039/c6sc05381e

**Published:** 2017-03-31

**Authors:** Guangyue Li, Peiyuan Yao, Rui Gong, Jinlong Li, Pi Liu, Richard Lonsdale, Qiaqing Wu, Jianping Lin, Dunming Zhu, Manfred T. Reetz

**Affiliations:** a Max-Planck-Institut für Kohlenforschung , Kaiser-Wilhelm-Platz 1 , 45470 , Mülheim an der Ruhr , Germany . Email: reetz@mpi-muelheim.mpg.de; b Fachbereich Chemie , Philipps-Universität , Hans-Meerwein-Strasse , 35032 Marburg , Germany; c National Engineering Laboratory for Industrial Enzymes , Tianjin Engineering Center for Biocatalytic Technology , Tianjin Institute of Industrial Biotechnology , Chinese Academy of Sciences , 32 Xi Qi Dao, Tianjin Airport Economic Area , Tianjin 300308 , People's Republic of China . Email: zhu_dm@tib.cas.cn ; Email: lin_jp@tib.cas.cn

## Abstract

A new directed evolution approach is presented to enhance the activity of an enzyme and to manipulate stereoselectivity by focusing iterative saturation mutagenesis (ISM) simultaneously on residues lining the entrance tunnel and the binding pocket. This combined mutagenesis strategy was applied successfully to the monoamine oxidase from *Aspergillus niger* (MAO-N) in the reaction of sterically demanding substrates which are of interest in the synthesis of chiral pharmaceuticals based on the benzo-piperidine scaffold. Reversal of enantioselectivity of Turner-type deracemization was achieved in the synthesis of (*S*)-1,2,3,4-tetrahydro-1-methyl-isoquinoline, (*S*)-1,2,3,4-tetrahydro-1-ethylisoquinoline and (*S*)-1,2,3,4-tetrahydro-1-isopropylisoquinoline. Extensive molecular dynamics simulations indicate that the altered catalytic profile is due to increased hydrophobicity of the entrance tunnel acting in concert with the altered shape of the binding pocket.

## Abstract 2

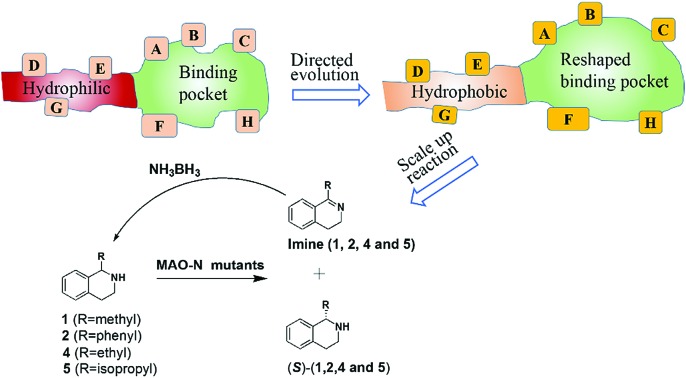
An efficient directed evolution strategy for enhancing activity and manipulating stereoselectivity of a monoamine oxidase is presented.

## Introduction

Directed evolution constitutes a versatile tool for engineering the catalytic profiles of enzymes, stereoselectivity, activity and substrate specificity being of particular interest in organic chemistry and biotechnology.^1^ Since screening is the labor-intensive step (bottleneck of directed evolution), efforts continue to focus on developing efficient mutagenesis strategies which enable the production of high-quality libraries requiring minimal analytical work.^1^,^2^ Iterative saturation mutagenesis (ISM)^3^ at sites lining the binding pocket is one of several approaches which deliver small and “smart” mutant libraries. Accordingly, sites labeled A, B, C, D, *etc.*, each comprising one or more residues, are first identified using X-ray data or homology models, followed by the generation of mutant libraries and respective screening. The best hit originating from one library is then used as a template for randomization at another site.

In many enzymes the active site is buried deeply within the core of the protein, which means that in order to access the binding pocket, substrates must pass first through the protein *via* an entrance tunnel.^4^ Unfortunately, sometimes it is difficult to identify the tunnel even when X-ray data is available. In some cases, the tunnel acts as a gate-keeper (molecular filter), which influences substrate selectivity and sometimes even activity as shown by mutagenesis experiments.^5^ Interest in identifying and understanding how these tunnels exert such control has grown steadily over the past several years,^4^ leading to the realization that in such cases tunnel optimization and binding pocket manipulation are both necessary for optimal catalytic performance. However, directed evolution studies encompassing mutagenesis at both types of sites are rare. A recent example is due to Hauer, Pleiss and coworkers, who performed separate saturation mutagenesis experiments at the active site and the entrance tunnel of a P450 monooxygenase, and subsequently combined the respective positive mutations.^6^

The purpose of the present study was to apply ISM with the aim of manipulating the size and polarity of an enzyme's entrance tunnel as well as the shape of its binding pocket. In principle, this can be attempted either sequentially or simultaneously. In the present study we decided to test the latter option as a new strategy in directed evolution. As will be seen, the substrate of interest is not accepted by the wild-type (WT) enzyme, which may be due to two different phenomena: the particular size and/or polarity of the tunnel prevents smooth substrate entry and product release, and/or the specific shape of the binding pocket is not conducive to catalytic turnover. If both effects operate, then focusing on them sequentially may fail to provide maximally improved variants.

In order to explore such a scenario, the monoamine oxidase from *Aspergillus niger* (MAO-N)^7^ was chosen as the model enzyme. In a series of previous studies by the Turner group devoted to the efficient deracemization of primary,^8^ secondary^9^ and tertiary amines^10^ as well as chiral heterocycles such as substituted pyrrolidines and tetrahydro-isoquinolines,^11^,^12^ this enzyme has already been subjected to directed evolution using mutator strain technology as well as saturation mutagenesis at the binding pocket and separately at the putative entrance to the active site.^12^ Although the crystal structure of wildtype (WT) MAO-N remains to be analyzed, X-ray structures of a 3-point mutant MAO-N-D3 and a 5-point variant MAO-N-D5 have been reported^13^ which helped in the interpretation of part of the data. The total length and size of the entrance tunnel were not explicitly defined.^12^,^13^ It should be pointed out that further protein engineering studies of MAO-N and of other MAOs have also appeared.^14^

## Results and discussion

**Scheme 1:**
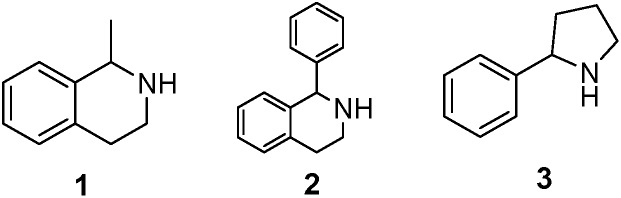
Model amino compounds **1**, **2** and **3**.

**Fig. 1:**
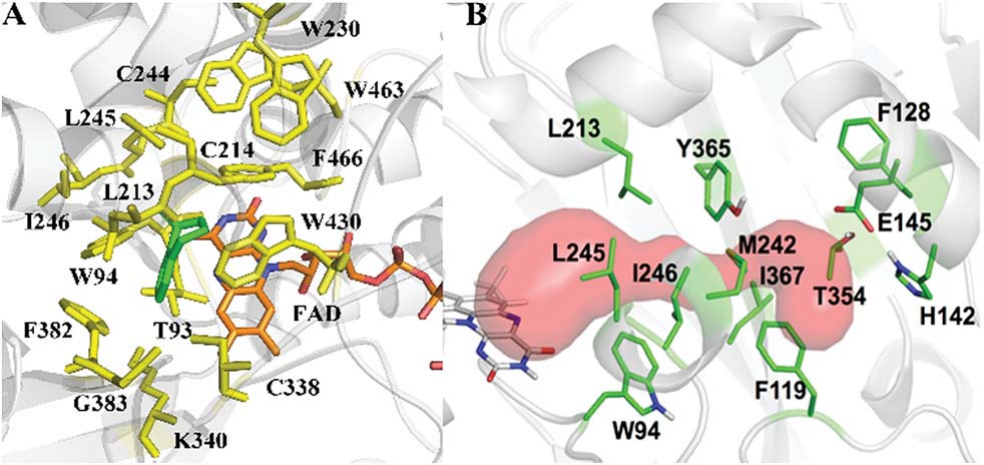
MAO-N residues chosen for saturation mutagenesis marked in the homology model, which was built using the crystal structure of MAO-N-D3 (PDB: 2VVL). (A) Active site mutation sites (yellow), selected on the basis of induced fit docking of amine **1** (green). (B) Residues surrounding the substrate access tunnel (red) likewise chosen for mutagenesis (shown in green).

As model compounds for assessing the activity of MAO-N mutants, we chose amines 1,2,3,4-tetrahydro-1-methylisoquinoline (**1**), 1,2,3,4-tetrahydro-1-phenylisoquinoline (**2**) and 2-phenyl-pyrroline (**3**) as shown in Scheme 1. The plan was to improve the activity towards amines **1** and **2** and to control stereoselectivity, these chiral compounds being of particular interest because the structural motifs occur in therapeutic drugs such as the proton pump inhibitor YH1885 15 and solifenacin,^16^ respectively. In our hands the compounds showed no activity in attempted WT MAO-N catalyzed reactions, in contrast to amine **3** which led to a slow but detectable transformation (ESI Scheme S1†). Therefore, we chose **3** as the model detector substrate for identifying functional enzyme residues that can then be used in ISM experiments for evolving variants that also accept substrates **1** and **2**. Upon creating a homology model based on MAO-N-D3 (N336S/M348K/I246M), the CAVER algorithm^17^ was applied with the aim of identifying the tunnel. In order to design an appropriate ISM scheme for MAO-N engineering, we considered two types of residues: amino acid positions lining the substrate **1** in the binding pocket [T93, W94, L213, C214, W230, C244, L245, I246, C338, K340, F382, G383, W430, W463 and F466], and those surrounding the extensive substrate access tunnel [W94, F119, F128, H142, E145, L213, M242, L245, I246, T354, Y365 and I367] (Fig. 1).

These two types of residues share four common residues [W94, L213, L245 and I246]. Consequently, we chose 23 residues for potential mutagenesis and split them into six groups as displayed in Table 1. Saturation mutagenesis at a 4-residue site using NNK codon degeneracy encoding all 20 canonical amino acids would require for 95% library coverage excessive screening (3 × 10^6^ transformants).^2^ Therefore, the reduced amino acid alphabet NDT was considered, encoding 12 amino acids (Phe, Leu, Ile, Val, Tyr, His, Asn, Asp, Cys, Arg, Ser, Gly), which requires the screening of about 6 × 10^4^ transformants for 95% library coverage.^2^ Although this is still a very high number, a previously developed easy-to-perform colorimetric on-plate assay^18^ for rapidly identifying thousands of active variants was applied in all mutagenesis experiments. The most active variants were subsequently tested as catalysts in Turner-type deracemization reactions.

**Table 1:** Grouping of the 23 chosen MAO-N residues into six randomization sites A, B, C, D, E and F and the NDT codes used in saturation mutagenesis. The green numbers denote “active site” positions, the red ones “tunnel” positions and purple ones “shared” positions

Randomization site	Code
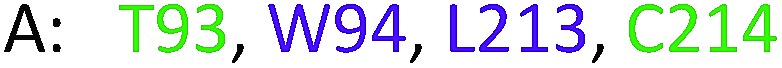	NDT
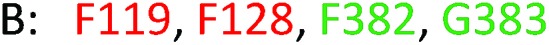	NDT
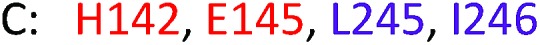	NDT
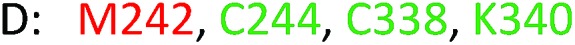	NDT
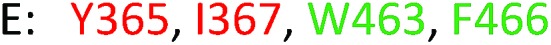	NDT
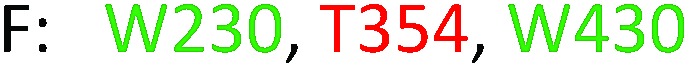	NDT

Libraries A, B, D, E and F contained a number of variants active for substrate **3** (ESI Table S1†), but only those in library F proved to be hits for substrates **1** and **2**, *e.g.*, W230R/W430C and W230I/T354S/W430R (ESI Tables S2 and S3†). Based on the result of sequence determination, we identified all activity related positions in sites A, B, D and E, namely F128, L213, C214, M242, Y365 and I367 (ESI Table S1†). Consequently, we regrouped these positions into sites G (F128, L213 and C214) and H (M242, Y365 and I367) (ESI Fig. S1a†). Thereafter, ISM was applied using the best variants as templates. The results are summarized in the ESI (Tables S2–S3 and Fig. S1b and c†).

The specific activities of WT MAO-N and the best mutants toward the model substrates are shown in Table 2, together with the results of deconvoluting variants of LG-F-B7. Improved variants were found in all cases. For example, the triple mutant LG-F-B7 (W230I/T354S/W430R) accepts the bulky substrate **2** with a specific activity of 0.3 U mg^–1^, which was further improved more than 2-fold by the introduction of two additional mutations (M242R/Y365V). Interestingly, in the case of substrate **1**, variant LG-I-D11 (W230R/W430C/C214L) shows a 6-fold increase in specific activity relative to LG-F-G6 (W230R/W430C), and also accepts the sterically demanding substrate **2**. The most striking result of the deconvolution experiments concerns the reaction of substrate **2**. Single mutant LG-F-B5 (T354S) shows no activity, but in concert with the double mutant LG-F-B5 (W230I/W430R) having a specific activity of 0.22 U mg^–1^, the respective triple mutant W230I/T354S/W430R displays a notable improvement (0.3 U mg^–1^). Thus, a strong cooperative effect is operating.^19^

**Table 2:** Specific activity (U mg^–1^) of WT MAO-N and evolved variants in addition to mutants LG-F-B6 and LG-F-B5 (marked in bold), which were created by deconvoluting LG-F-B7

Entry	Mutations	**1**	**2**	**3**
WT MAO-N		0	0	0.13 ± 0.01
LG-F-G6	W230R/W430C	0.108 ± 0.001	0.23 ± 0.02	0.04 ± 0.002
LG-I-D11	W230R/W430C/C214L	0.66 ± 0.01	0.52 ± 0.05	0.23 ± 0.02
LG-F-B7	W230I/T354S/W430R	0.02 ± 0.001	0.30 ± 0.01	0.02 ± 0.003
LG-J-B4	W230I/T354S/W430R/M242R/Y365V	0.018 ± 0.001	0.67 ± 0.03	0.03 ± 0.003
**LG-F-B6**	**W230I/W430R**	**0.014 ± 0.001**	**0.22 ± 0.02**	**0.016 ± 0.004**
**LG-F-B5**	**T354S**	**0.025 ± 0.001**	**0**	**0.09 ± 0.01**

The kinetic parameters of the best MAO-N variants (LG-J-B4 and LG-I-D11), mutant LG-F-B7 and deconvolutant LG-F-B6 toward substrates **1** and **2** were obtained by measuring the initial velocities of the enzymatic reaction and curve-fitting according to the Michaelis–Menten equation. In the cases of LG-I-D11 (for amine **1**) and LG-J-B4 (for amine **2**) the *V*_max_ proved to be 1.12 U mg^–1^ and 0.82 U mg^–1^ (Fig. S11†), respectively. The data for the catalytic rate (*k*_cat_) and catalytic efficiency (*k*_cat_/*K*_m_) are summarized in Table 3. In the case of substrate **1**, the previously evolved mutant Asn336Ser/Ile246Met was reported to have moderate activity (*k*_cat_ = 6.00 min^–1^; *k*_cat_/*K*_m_ = 19.35 min^–1^ mM^–1^).^9^ In the present work, variant LG-I-D11 (W230R/W430C/C214L) shows a 10- and 2.8-fold improvement in *k*_cat_ and *k*_cat_/*K*_m_, respectively. Additionally, the catalytic efficiency for substrate **2** increases from LG-F-B6 and LG-F-B7 to LG-J-B4. To the best of our knowledge, the kinetic parameters of previous MAO-N variants in the reaction of substrate **2** have not been reported to date.

**Table 3:** Kinetic characterization of the best MAO-N variants, mutant LG-F-B7 and deconvolutant LG-F-B6

Enzyme	Substrate	*K*_m_ (mM)	*k*_cat_ (min^–1^)	*k*_cat_/*K*_m_ (min^–1^ mM^–1^)
LG-I-D11	**1**	1.12 ± 0.09	61.77 ± 1.41	55.15
LG-J-B4	**2**	1.25 ± 0.06	46.02 ± 0.60	36.81
LG-F-B7	**2**	1.42 ± 0.07	37.40 ± 0.72	26.34
LG-F-B6	**2**	1.96 ± 0.12	21.35 ± 0.43	10.89

**Fig. 2:**
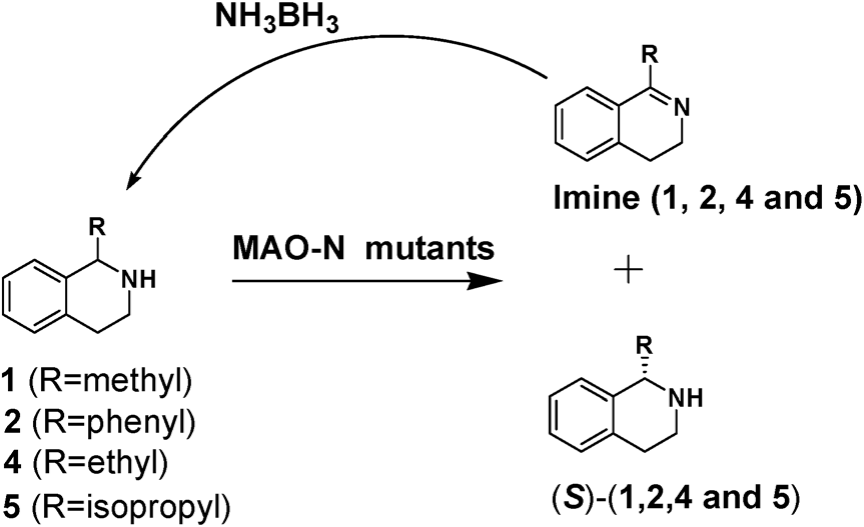
Deracemization of racemates **1**, **2**, **4** and **5** by employing a cyclic sequence of enantioselective oxidation with MAO-N mutants (LG-I-D11 and LG-J-B4) and non-selective reduction with NH_3_·BH_3_.

We then applied the Turner-deracemization technique^8^–^12^,^18^ to substrates **1** and **2** using recombinant cells of MAO-N mutants LG-I-D11 and LG-J-B4, respectively. This procedure employs a cyclic sequence of enantioselective oxidation with MAO-N mutants (LG-I-D11 and LG-J-B4) and non-selective chemical reduction using NH_3_·BH_3_ as shown in Fig. 2. The deracemized products were recovered in 73% and 86% isolated yields of (*S*)-**1** and (*S*)-**2**, respectively, each with high enantiomeric excess (>99% and 93.4%) (Table 4, Fig. S12–S15†). This is a significant result, because thus far 1,2,3,4-tetrahydro-1-methylisoquinoline (**1**) could be obtained only as the (*R*)-enantiomer, as shown by catalysis using the known variant MAO-N D5.^9^,^20^ Therefore, reversal of enantioselectivity has been achieved by introducing the present point mutations. In order to test the generality of the mutant LG-I-D11, deracemization was also carried out for 1,2,3,4-tetrahydro-1-ethylisoquinoline (**4**) and 1,2,3,4-tetrahydro-1-isopropylisoquinoline (**5**). The deracemized products were recovered in 80% and 81% isolated yields of (*S*)-**4** and (*S*)-**5**, respectively, also with high enantiomeric excess (>99%) (Table 4, Fig. S16–S19†). Compounds *rac*-**4** and *rac*-**5** have not been previously subjected to MAO-N-catalyzed deracemization, in contrast to *rac*-**2** (providing (*R*)-**2** during deracemization).^9^,^20^ Tetrahydroisoquinolines (THIQs) belong to a family of biologically active molecules, including natural alkaloids and important pharmaceutical products.^21^

**Table 4:** Scaled-up deracemization of tetrahydroisoquinolines using best mutants LG-I-D11 and LG-J-B4 leading to (*S*)-products

Enzyme	Substrate	ee (%)	Isolated yield (%)
LG-I-D11	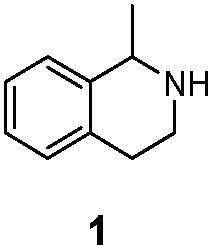	>99% (*S*)	73%
LG-I-D11	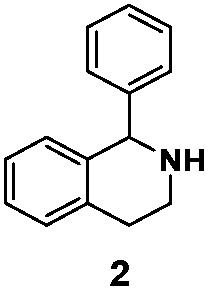	80% (*S*)	81%
LG-J-B4	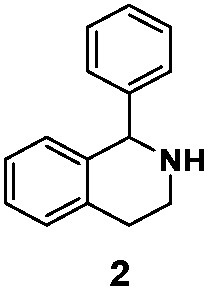	93.4% (*S*)	86%
LG-I-D11	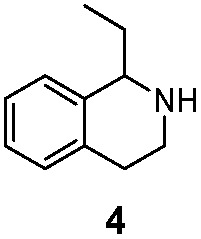	>99% (*S*)	80%
LG-I-D11	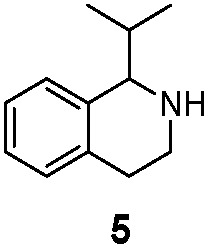	>99% (*S*)	81%

**Scheme 2:**
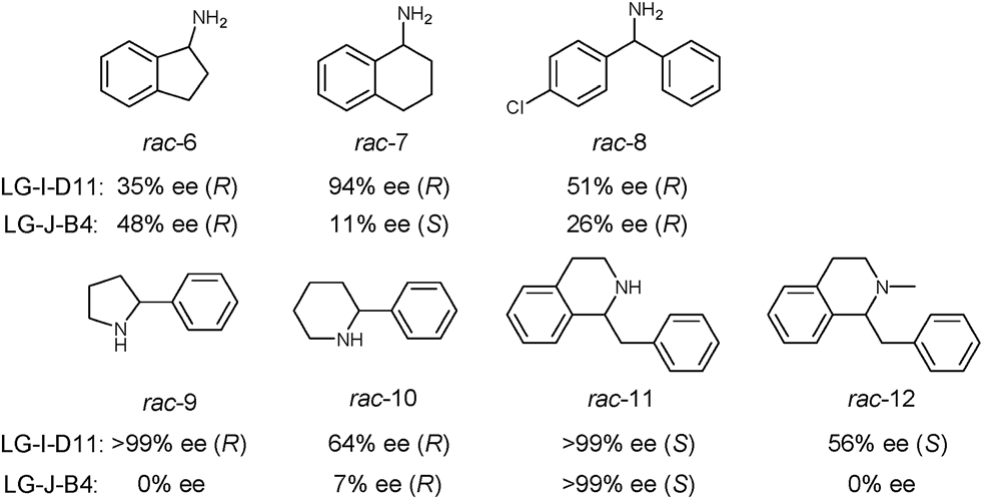
Deracemization of a panel of amine substrates using best mutants LG-I-D11 and LG-J-B4.

In order to further explore the substrate specificity, several structurally diverse amines were subjected to MAO-N catalysis for deracemization with LG-I-D11 and LG-J-B4 in analytical scale reactions, respectively (Scheme 2, Fig. S20–31 and Table S5†) without carrying out additional mutagenesis experiments. In some but not in all cases excellent results were obtained. The deracemization of amines **7**, **9** and **11** with L-I-D11 resulted in 94% ee, 99% ee and 99% ee, respectively, demonstrating high activity and enantioselectivity. In these cases reversal of enantioselectivity relative to the Turner results was not observed.^8^–^10^,12b,^22^ Relative to LG-I-D11, a higher ee-value (48%) was obtained for amine **6** by deracemization with mutant LG-J-B4. Interestingly, LG-J-B4 showed reversed stereoselectivity for amine **7** compared with LG-I-D11. This indicates that it should be possible to invert stereoselectivity in general, but such a task would require further mutational studies.

**Fig. 3:**
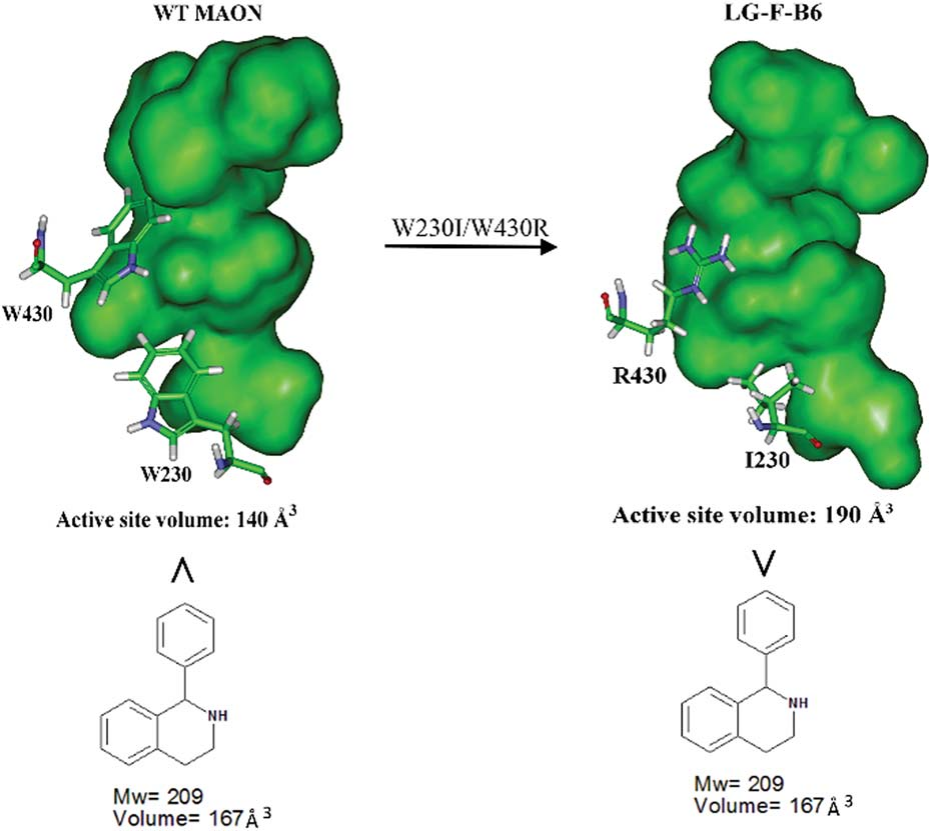
Comparison of the active site volumes of WT MAO-N and variant LG-F-B6, computed by application of Accelrys Discovery Studio software 4.1 in the search for enzyme cavities.

In order to gain insight into the source of enhanced catalyst activity, the ISM evolutionary process of MAO-N was analyzed using compound **2** as substrate. We addressed this problem by applying the Accelrys Studio 4.1 software for identifying cavities and assessing their volume and molecular dynamics (MD) simulations for characterizing the altered nature of the tunnel. In the first step going from WT MAO-N to variant LG-F-B7, which was discovered in library F, three point mutations (W230I/T354S/W430R) were introduced, W230I (active site), T354S (tunnel), and W430R (active site). The two deconvolutants LG-F-B6 (W230I/W430R) and LG-F-B5 (T354S) (Table 2) allowed us to identify the respective mutational effects. The catalytic profile of deconvolutant LG-F-B6 indicates that mutations W230I/W430R have changed the size and thus shape of the active site pocket so that substrate **2** is accepted with notable activity, but not in the case of the single site mutation LG-F-B5 (T354S) which occurs at the beginning of the entrance tunnel. When comparing the shape of the computed active site pocket of WT MAO-N with that of LG-F-B6 (Fig. 3), it becomes clear that in the latter variant the exchange of Trp (W) at positions 230 and 430 by sterically smaller amino acids Ile and Arg cause severe geometric changes. The concomitant increase in volume of the active site pocket allows the binding of large bulky steric substrates such as **2** with two aryl moieties (volume = 167 Å^3^).

**Fig. 4:**
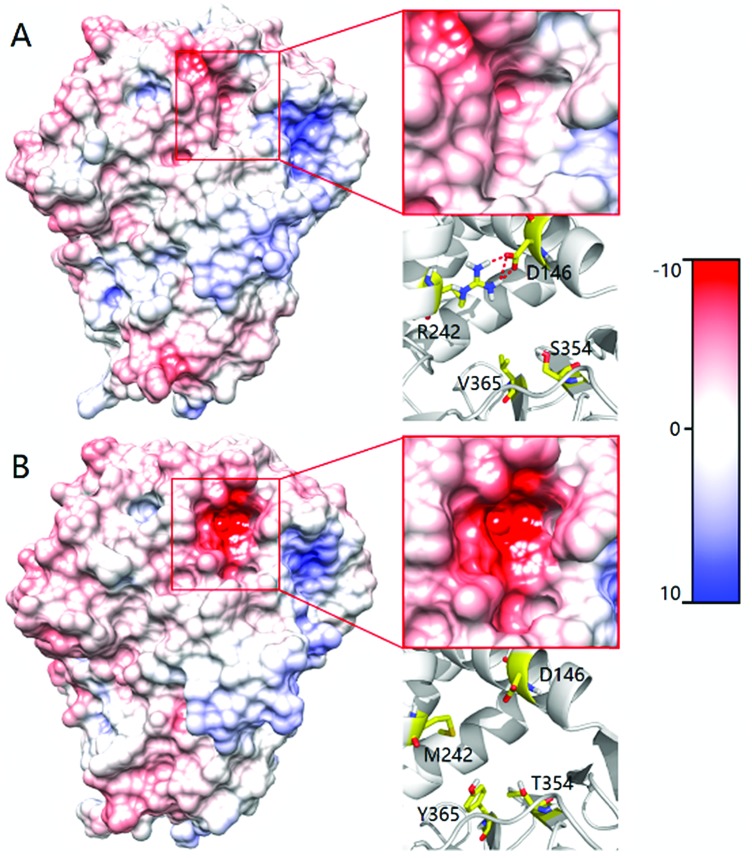
Comparison of the tunnel surface potential of LG-F-B4 (A) and LG-J-B6 (B). Different colors denote different levels of polarity, deeper red coloring denoting increasing polarity.

In order to shed light on the further enhanced catalyst activity upon going from LG-F-B6 to LG-J-B4, MD simulations were performed in the absence of **2**. This allowed the visualization of representative conformations of variants LG-F-B4 and LG-J-B6 without any bias arising from interactions with the substrate. A representative conformation was selected after clustering of the phase space sampled from the 50 ns MD trajectory. The conformational change of tunnels of LG-F-B4 and LG-J-B6 is described in Fig. 4. The side-chain of M242R forms a hydrogen bond with the oxygen atom of residue D146 which reduces the polarity of the tunnel. The mutation Y365V results in a similar effect at the tunnel entrance and exit (Fig. 4A). In contrast, residues D146 and Y365 do not undergo similar interactions and consequently do not contribute to a reduction of the polarity in the tunnel (see Fig. 4B). Overall, a decrease in polarity makes it easier for hydrophobic substrates and products to enter and exit the enzyme, thereby increasing the catalytic activity of LG-J-B4.^23^ The respective engineered tunnels of LG-F-B4 and LG-J-B6 are shown in Fig. S32 (ESI†). It should be pointed out that in the present case the tunnel volume has not increased significantly upon mutagenesis, which means that the polarity change upon going from LG-J-B6 to LG-F-B4 constitutes the determining factor.

In order to throw light on possible reasons for reversed stereoselectivity of LG-I-D11 in the case of substrate **1**, two structural models were built for variant LG-I-D11 harboring (*R*)-**1** and (*S*)-**1**, respectively, using the Autodock 4.2 program.^24^ Based on the previously proposed and generally accepted mechanism,^25^ docking conformations were selected. The binding models of the respective complexes (*R*)-**1** and (*S*)-**1** to LG-I-D11 are actually quite similar (Fig. S33†). The benzene ring of substrate **1** is deeply embedded in the hydrophobic area of the binding site, and the amino-moiety points toward the FAD. This computational result is consistent with the crystal structure of the MAO-N-D5-proline complex (2VVM). It suggests that our LG-I-D11-(*R*)-**1** and LG-I-D11-(*S*)-**1** models derived from docking are reliable and can be used for further MD simulations. Since transformations of both (*R*)- and (*S*)-**1** are catalyzed by variant LG-I-D11, a distance constraint (2.5 Å, 50 kcal mol^–1^) between the coordinating N-atom of FAD and the H-atom of the stereogenic C-atom in the two enantiomers was applied in the first 5 ns of MD, thereby simulating the induced-fit process of substrate binding. This corresponds to a pose close to the transition state. The molecular mechanics generalized Born surface area (MM-GBSA) method implemented in AMBER16 was used to calculate binding free energies of the LG-I-D11-complexes of (*R*)- and (*S*)-**1** after 5 ns MD simulations. Table S6† shows that the MM-GBSA predicted binding free energy (GBTOT) for the (*R*)- and (*S*)-complexes amount to –23.69 kcal mol^–1^ and –16.70 kcal mol^–1^, respectively. It is notable that the computed binding energy of LG-I-D11-(*R*)-**1** is approximately 6.97 kcal mol^–1^ smaller than the LG-I-D11-(*S*)-**1** complex. We are aware of the necessity to compute transition state energies by quantum mechanical methods, but this was outside the realm of the present study. At this stage we point to the study of Sherman and coworkers who examined the relationship between binding affinity and catalytic activity.^26^

**Fig. 5:**
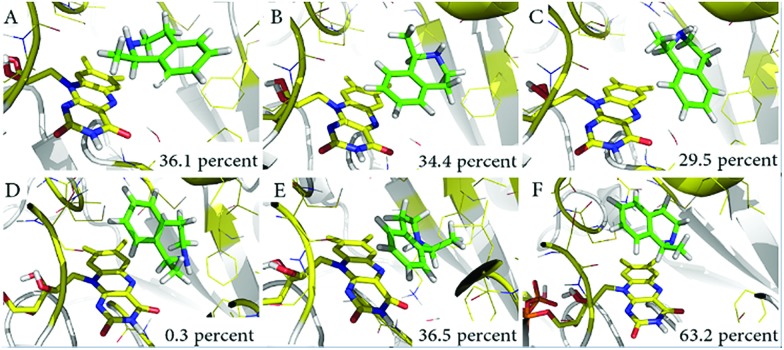
Representative conformations of three clusters in MD simulations. (A) Catalytically active conformations of LG-I-D11-(*R*)-**1**; (B) and (C) show conformations which severely restrict catalytic activity; (D) catalytically active conformations of LG-I-D11-(*S*)-**1**; (E) and (F) show conformations which also severely restrict catalytic activity.

With the aim of exploring the conformational changes at the catalytic active site, the entire systems were equilibrated without any constraints by MD for 50 ns at 300 K. The MD trajectory was aligned and clustered into three clusters for each system based on the backbone atoms of variant LG-I-D11 to identify the most representative conformations of the sampled phase space. The representative structures of catalytic domain from three highly clustered conformations of MD simulation trajectory are shown in Fig. 5. The frequency of conformations in each cluster was calculated in order to explore the possibility of emerging active conformations. The results of the simulations show that in the LG-I-D11-(*R*)-**1** complex, the interatomic distance between the N-atom of FAD and the H-atom of the stereogenic carbon in (*R*)-**1** is constant at a distance of ∼2.1 Å (Fig. 5A), a feature which supports catalysis. We also found a similar phenomenon in the LG-I-D11-(*S*)-**1** complex, in this case the distance being ∼2.2 Å (Fig. 5D), but the respective frequencies are dramatically different: LG-I-D11-(*R*)-**1** complex (36.1%) *versus* LG-I-D11-(*S*)-**1** complex (0.3%). Other clusters are characterized by long distances as shown in Fig. 5B (4.9 Å), 5C (4.4 Å), 5E (3.6 Å) and 5F (3.3 Å). Based on the analysis of the distance between FAD and substrate in multiple monoamine oxidases which reveal distances of less than 2.5 Å, we conclude that these are inactive conformations. Moreover, during the MD simulation of the (*R*)-**1**-complex, the original conformation is maintained (Fig. S34†). This computational result is in line with the experimentally observed stereoselectivity (Table 4).

## Conclusions

We have successfully applied saturation mutagenesis for the first time simultaneously at residues surrounding the tunnel and lining the binding pocket of an enzyme in order to enhance activity and to manipulate stereoselectivity. For optimal results, iterative saturation mutagenesis (ISM) was invoked. This combined strategy is illustrated using the monoamine oxidase from *Aspergillus niger* (MAO-N) as the enzyme and structurally different amines as substrates. When deracemizing 1,2,3,4-tetrahydro-1-methylisoquinoline (**1**) on a preparative scale, reversal of enantioselectivity was observed, which means that the new MAO-N variant LG-I-D11 allows for the first time access to the (*S*)-enantiomer. Moreover, variant LG-I-D11 also showed good activity in the preparation of (*S*)-1,2,3,4-tetrahydro-1-ethylisoquinoline and (*S*)-1,2,3,4-tetrahydro-1-isopropylisoquinoline on a preparative scale. The best variants LG-I-D11 and LG-J-B4 were further used for deracemization of a panel of structurally diverse amines in analytical scale reactions, some excellent results being obtained with the same enantioselectivity as with the Turner results.^8^–^10^,12b,^22^ However, LG-J-B4 showed reversed stereoselectivity for amine **7** compared with the performance of variant LG-I-D11. This indicates that it should be possible to invert stereoselectivity in general, but such a task would require further mutational studies.

A reasonable model for explaining the increase in activity was developed on the basis of the computed active site volumes and analysis of tunnel polarity from MD simulations. The reversed stereoselectivity was also explored by MD simulations, specifically by considering different binding energies and frequency of catalytically active conformations. QM/MM computations would need to be performed for further insight.

The mutagenesis strategy described herein deserves comment. Variant LG-I-D11 is characterized exclusively by “active site” mutations (W230R/W430C/C214L), which may suggest that saturation mutagenesis at sites lining the binding pocket would have sufficed in reaching all goals of the study. However, such a conclusion cannot be upheld when viewing other data. In the case of the hydrophobic substrate **2** this triple is a poor catalyst, showing only moderate enantioselectivity (80% ee (*S*), Table 4). In contrast, variant LG-J-B4 (W230I/T354S/W430R/M242R/Y365V) with three point mutations occurring at tunnel sites and two lining the binding pocket is more active and enantioselective (93.4% ee (*S*)).

It is also instructive to compare the catalytic profiles of the three variants LG-J-B4 (3 tunnel and 2 active site mutations), LG-F-B7 (1 tunnel and 2 active site mutations) and LG-F-B6 (no tunnel mutations, only 2 active site mutations). In the reaction of the bulky and hydrophobic substrate **2**, it can be seen that catalytic rate (*k*_cat_) and catalytic efficiency (*k*_cat_/*K*_m_) decrease sharply in this order (Table 3). The evolved change in tunnel polarity supports substrate entry and product departure. We conclude that in contrast to the successful simultaneous mutagenesis strategy described herein, engineering the entrance tunnel polarity and the shape of the binding pocket sequentially involves some uncertainty and may not be as successful. We expect the combined ISM mutagenesis approach to be applicable to other enzymes in which not only the shape of the binding pocket plays a role, but also the characteristics of the entrance tunnel which include such properties as polarity, shape and size.

## Supplementary Material

Supplementary information
