# Correction: Simultaneous engineering of an enzyme’s entrance tunnel and active site: the case of monoamine oxidase MAO-N

**DOI:** 10.1039/c7sc90029e

**Published:** 2017-05-16

**Authors:** Guangyue Li, Peiyuan Yao, Rui Gong, Jinlong Li, Pi Liu, Richard Lonsdale, Qiaqing Wu, Jianping Lin, Dunming Zhu, Manfred T. Reetz

**Affiliations:** a Max-Planck-Institut für Kohlenforschung , Kaiser-Wilhelm-Platz 1 , 45470 , Mülheim an der Ruhr , Germany . Email: reetz@mpi-muelheim.mpg.de; b Fachbereich Chemie , Philipps-Universität , Hans-Meerwein-Strasse, Marburg , 35032 , Germany; c National Engineering Laboratory for Industrial Enzymes , Tianjin Engineering Center for Biocatalytic Technology , Tianjin Institute of Industrial Biotechnology , Chinese Academy of Sciences , 32 Xi Qi Dao, Tianjin Airport Economic Area , Tianjin 300308 , People’s Republic of China . Email: zhu_dm@tib.cas.cn

## Abstract

Correction for ‘Simultaneous engineering of an enzyme’s entrance tunnel and active site: the case of monoamine oxidase MAO-N’ by Guangyue Li *et al.*, *Chem. Sci.*, 2017, 8, 4093–4099.



## 

The authors regret that there are instances in the work where the mutants and one compound are mislabelled. These are described correctly below:

On page 4095, in the text discussing the single, double and triple mutants, the double mutant is mislabelled as LG-F-B5 and should instead read:

Single mutant LG-F-B5 (T354S) shows no activity, but in concert with the double mutant LG-F-B6 (W230I/W430R) having a specific activity of 0.22 U mg^–1^, the respective triple mutant W230I/T354S/W430R displays a notable improvement (0.3 U mg^–1^). Thus, a strong cooperative effect is operating.^19^

On page 4095, in the text discussing the deracemization of the compounds **1–5**, the racemic compound **1** is mislabelled as *rac*-**2** and should instead read:

Compounds *rac*-**4** and *rac*-**5** have not been previously subjected to MAO-N-catalyzed deracemization, in contrast to *rac*-**1** (providing (*R*)-**1** during deracemization).^9,20^

On pages 4096–4097, in the paragraph discussing the catalytic activity when moving from LG-F-B6 to LG-J-B4, the terms LG-F-B6 and LG-J-B4 are incorrectly displayed. The corrected text is included below:

In order to shed light on the further enhanced catalyst activity upon going from LG-F-B6 to LG-J-B4, MD simulations were performed in the absence of **2**. This allowed the visualization of representative conformations of variants LG-J-B4 and LG-F-B6 without any bias arising from interactions with the substrate. A representative conformation was selected after clustering of the phase space sampled from the 50 ns MD trajectory. The conformational change of tunnels of LG-J-B4 and LG-F-B6 is described in Fig. 4. The side-chain of M242R forms a hydrogen bond with the oxygen atom of residue D146 which reduces the polarity of the tunnel. The mutation Y365V results in a similar effect at the tunnel entrance and exit (Fig. 4A). In contrast, residues D146 and Y365 do not undergo similar interactions and consequently do not contribute to a reduction of the polarity in the tunnel (see Fig. 4B). Overall, a decrease in polarity makes it easier for hydrophobic substrates and products to enter and exit the enzyme, thereby increasing the catalytic activity of LG-J-B4.^23^ The respective engineered tunnels of LG-J-B4 and LG-F-B6 are shown in Fig. S32 (ESI†). It should be pointed out that in the present case the tunnel volume has not increased significantly upon mutagenesis, which means that the polarity change upon going from LG-F-B6 to LG-J-B4 constitutes the determining factor.

In the caption of Fig. 4, the terms LG-J-B4 and LG-F-B6 are displayed incorrectly and should read:

**Fig. 4.** Comparison of the tunnel surface potential of LG-J-B4 (A) and LG-F-B6 (B). Different colors denote different levels of polarity, deeper red coloring denoting increasing polarity.

The Royal Society of Chemistry apologises for these errors and any consequent inconvenience to authors and readers.

