# Supplementary material for: Simultaneous engineering of an enzyme's entrance tunnel and active site: the case of monoamine oxidase MAO-N
Source: Chem Sci. 2017 Mar 31;8(5):4093–9. doi: 10.1039/c6sc05381e (PMC6099926; doi:10.1039/c6sc05381e)
Supplement: Supplementary file 1 [file SC-008-C6SC05381E-s001.pdf]

Electronic Supplementary Material (ESI) for Chemical Science.
This journal is © The Royal Society of Chemistry 2017

Simultaneous engineering of an enzyme's entrance tunnel and active site: The case of monoamine oxidase MAO-N

Guangyue Li[a, b,§], Peiyuan Yao[c, §], Rui Gong[c,§], Jinlong Li[c], Pi Liu[c], Richard Lonsdale[a, b], Qiaqing Wu[c], Jianping Lin [c,*], Dunming Zhu[c,*] and Manfred T. Reetz[a, b,*]

[a.] Max-Planck-Institut für Kohlenforschung, Kaiser-Wilhelm-Platz 1, 45470, Mülheim an der Ruhr, Germany.
E-mail: reetz@mpi-muelheim.mpg.de.

[b.] Fachbereich Chemie, Philipps-Universität, Hans-Meerwein-Strasse, 35032 Marburg, Germany.

[c.] National Engineering Laboratory for Industrial Enzymes and Tianjin Engineering Center for Biocatalytic Technology, Tianjin Institute of Industrial Biotechnology, Chinese Academy of Sciences, 32 Xi Qi Dao, Tianjin Airport Economic Area, Tianjin 300308, People's Republic of China.

Content

Materials and chemicals 4

Method 4

- Construction of the expression plasmid of WT MAO-N 4
- The selection of mutagenesis residues 4
- PCR based methods for library construction of MAO-N 4
- Primer design and library creation of MAO-N 4
- Screening of the mutant libraries 5
- Activity assay 5
- Expression and purification of WT MAO-N and positive mutants 5
- Determination of kinetic parameters 5
- Preparative scale deracemization of substrates using recombinant cells of MAO-N mutants 5
- General procedure for the deracemization 6
- Modelling and molecular dynamics simulation 7

Supplementary Schemes， Figures and Tables 7

- Scheme S1. Oxidation of amine 3 catalyzed by the monoamine oxidase MAO-N 7
- Figure S1. The directed evolution strategy of MAO-N toward amines **1** and **2** 9

Figure S2. Primer design and library creation of WT MAO-N for library A. NDT were used as the building blocks. 9

Figure S3. Primer design and library creation of WT MAO-N for library B. NDT were used as the building blocks. 9

Figure S4. Primer design and library creation of WT MAO-N for library C. NDT were used as the building blocks. 9

Figure S5. Primer design and library creation of WT MAO-N for library D. NDT were used as the building blocks. 10

Figure S6. Primer design and library creation of WT MAO-N for library E. NDT were used as the building blocks. 10

Figure S7. Primer design and library creation of WT MAO-N for library F. NDT were used as the building blocks. 10

Figure S8. Primer design and library creation of WT MAO-N for library G. NDT were used as the building blocks. 10

Figure S9. Primer design and library creation of WT MAO-N for library H. NDT were used as the building blocks. 11

Figure S10. SDS-page of purified WT MAO-N and relative mutants. M: Marker, 1: WT MAON, 2: LG-F-B7, 3: LG-J-B4, 4: LG-F-G6, 5: LG-I-D11, 6: LG-F-B6 and 7: LG-F-B5. 11

Figure. S11 Kinetic analysis of MAON mutants (LG-I-D11 for substrate 1, LG-J-B4, LG-F-B7 and LG-F-B6 for substrate 2)..........11

Figure S12. HPLC spectra of racemic 1, the product of variants LG-I-D11 ((*S*)-1) and MAO-N D5 (58.8% ee)..........12

Figure S13. HPLC spectra of racemic 2 and the product of variants LG-J-B4 ((*S*)-2) and LG-I-D11 ((*S*)-2). 12

Figure S14. NMR spectra of the product (*S*)-1. 13

Figure S15. NMR spectra of the product (*S*)-2. 14

Figure S16. HPLC spectra of racemic 4 and the product (*S*)-4. 14

Figure S17. HPLC spectra of racemic 5 and the product (*S*)-5. 14

Figure S18. NMR spectra of the product (*S*)-4. 15

Figure S19. NMR spectra of the product (*S*)-5. 15

Figure S20. HPLC spectra of racemic 6 and the product of LG-J-B4............................... 16

Figure S21. HPLC spectra of racemic 6 and the product of LG-I-D11............................. 16

Figure S22. HPLC spectra of racemic 7 and the product of LG-J-B4............................... 17

Figure S23. HPLC spectra of racemic 7 and the product of LG-I-D11............................. 17

Figure S24. HPLC spectra of racemic 8 and the product of LG-J-B4............................... 18

Figure S25. HPLC spectra of racemic 8 and the product of LG-I-D11............................. 18

Figure S26. HPLC spectra of racemic 9 and the product of LG-I-D11............................. 19

Figure S27. HPLC spectra of racemic 10 and the product of LG-J-B4............................. 19

Figure S28. HPLC spectra of racemic 10 and the product of LG-I-D11........................... 19

Figure S29. HPLC spectra of racemic 11 and the product of LG-J-B4............................. 20

Figure S30. HPLC spectra of racemic 11 and the product of LG-I-D11........................... 20

Figure S31. HPLC spectra of racemic 12 and the product of LG-I-D11........................... 21

Figure S32. The green balls represent the tunnels of LG-F-B4 (A) and LG-J-B6 (B).......... 21

Figure S33. The superimposition of the binding models of the respective complexes (*R*)-1 and (*S*)-1 to LG-I-D11. 22

Figure S34. The rmsd compute of LG-I-D11-*R*1 (A) and LG-I-D11-*S*1 (B). 22

Table S1. Results of screening libraries of MAO-N for amine 3 using code NDT. 22

Table S2. Results of screening libraries of MAO-N for amine 1 using code NDT. 23

Table S3. Results of screening libraries of MAO-N for amine 2 using code NDT. 24

Table S4. List of primers for MAO-N libraries A-H. 26

Table S5 HPLC columns, conditions and retention times. 27

Table S6: The MM-GBSA predicted binding free energy for LG-I-D11-R1 and LG-I-D11-S1. 27

References 28

Materials and chemicals

KOD Hot Start DNA Polymerase was obtained from Novagen. Restriction enzyme *Dpn* I was bought from NEB. The oligonucleotides were synthesized by Life Technologies. Plasmid preparation kit was ordered from Zymo Research, and PCR purification kit was bought from QIAGEN. DNA sequencing was conducted by GATC Biotech. All commercial chemicals were purchased from Sigma-Aldrich, Tokyo Chemical Industry (TCI) or Alfa Aesar.

Method

Construction of the expression plasmid of WT MAO-N

The full length WT MAO-N gene was PCR amplified from the synthetic WT MAO-N gene with 6-His tag purchased from GENEWIZ, Inc (www.genewiz.com) by the following primers (forward primer: 5’-CATGCATGCCATGGGCACCAGCCGCGACGGCTATCAGTGGA -3’; reverse primer: 5’-CGCCGCGGATCCTCAGTGGTGGTGGTGGTGGTGCAGGC-3’). The recombinant WT MAO-N gene was generated by the insertion of the PCR product into pRSF-Duet-1 vector via *Nco* I and *Bam*H I sites with an C-terminal 6-His tag and verified by sequencing.

The selection of mutagenesis residues

The homology model of WT MAO-N was built using the X-ray crystal structure of the D3 variant of MAO-N from *Aspergillus Niger* (PDB: 2VVL) as template using the modeling package in the Accelrys Discovery Studio 4.1. Then substrate was modeled into the active center. All the residues lining the active center were selected to be within 5 Å of the docked substrate. The substrate access tunnel was determined using the Caver program on clustered structures of the homology model, the minimum probe radius was 0.7 Å and the coordinate of starting point was X:-0.874, Y:-16.474 and Z: -31.969. The residues surrounding the substrate access tunnel were selected for mutagenesis.

PCR based methods for library construction of MAO-N

Libraries were constructed using the Over-lap PCR and megaprimer approach with KOD Hot Start polymerase. 50 µL reaction mixtures typically contained 30 µL water, 5 µL KOD hot start polymerase buffer (10×), 3 µL 25 mM $MgSO_4$, 5 µL 2 mM dNTPs, 2.5 µL DMSO, 0.5 µL (50~100 ng) template DNA, 100 µM primers Mix (0.5 µL each) and 0.5 µL KOD hot start polymerase. The PCR conditions for short fragment: 95 °C 3 min, (95 °C 30 sec, 56 °C 30 sec, 68 °C 40 sec) × 30 cycles, 68 °C 120 sec. For mega-PCR: 95 °C 3 min, (95 °C 30 sec, 60 °C 30 sec, 68 °C 5 min 30 sec) × 28 cycles, 68 °C 10 min. The PCR products were analyzed on agarose gel by electrophoresis and purified using a Qiagen PCR purification kit. 2 µL NEB CutSmart™ Buffer and 2 µL *Dpn* I were added in 50 µL PCR reaction mixture and the digestion was carried out at 37 °C for 7 h. After *Dpn* I digestion, the PCR products 1.5 µL were directly transformed into electro-competent *E. coli* BL21(DE3) to create the final library for Quick Quality Control and screening.

Primer design and library creation of MAO-N

Primer design and library construction depend upon the particular amino acid chosen, and in the case of MAO-N involves twenty-three residues, which were divided into six groups (Fig. S2-Fig. S7): 1) Amplification of the short fragments of MAO-N using mixed primers F1/R1, F2/R2, F3/R3, F4/R4 and F5/R5 for Library A, B, C, D and E respectively. Amplification of the short fragments of MAO-N using mixed primers F6/R6 and F7/R7 for Library F; 2) Over-lap PCR using the PCR products of F6/R6 and F7/R7 as template and mixed primers F6/R7; 3) Amplification of the whole plasmid pRSF-MAO-N using the PCR products of F1/R1, F2/R2, F3/R3, F4/R4, F5/R5 and over-lap PCR product of step2 as megaprimers, leading to the final variety plasmids for library generation. Other Libraries (Figure S8-S9) were created using the same procedure as mentioned above and all the primers used are listed in Table S4. The PCR products were digested by *Dpn* I and transformed into electro-competent *E. coli*BL21 (DE3) to create the library for screening. The transformants were plated on HiBond-C Extra membranes

placed on LB agar plates containing 50 µg/ml kanamycin and 0.5 mM IPTG, respectively. The plates were incubated at 30°C for 24 hours. The resulting first round of mutant libraries were screened with the **1**, **2** or **3** as substrate as described below.

Screening of the mutant libraries

The plate assay method described by Turner group was followed. Specifically, the Hi-BondC Extra membranes containing the clones were pulled from the LB agar plates and kept at -20°C for 24 hours to lyse the cells. The membranes were incubated at room temperature for 12 hours with an assay mixture containing 1 tablet of diaminobenzidine (DAB), 1 ml of potassium phosphate buffer (1 M, pH 7.0), 20 µL of screening substrate (100 mM) solution, 30 µL of horseradish peroxidase (5 mg/mL), 10 mL of 2% agarose and water up to 20 mL. Positive clones were picked and inoculated on LB agar plates (50 µg/ml kanamycin) every two hours. The selected positive clones were subjected to activity measurement (see below activity assay) and the mutations were identified by DNA sequencing and amino acid sequence verification.

Activity assay

The enzyme specific activities were assayed using a SPECTRAMAX M2e (MD, USA) at 30°C. Initial rates of the reaction were measured via the absorbance of a dye (ε = 29.4 mmol L^{-1} cm^{-1}) at 510 nm , which was produced by the action of horseradish peroxidase with the liberated hydrogen peroxide from the oxidation of the amine by MAO-N or variants, 4-aminoantipyrine, and 2,4,6-tribromo-3-hydroxybenzoicacid. The assay mixture (0.2 mL total volume) contained 174 µL of phosphate buffer (50 mM, pH 7.4), 2 µL of a 2, 4, 6-tribromo-3-hydroxybenzoic acid stock solution (20 mg/mL in DMSO), 2 µL of 4-aminoantipyrine stock solution (15 mg/mL in H2O), 2 µL of an amine stock solution (0.5 M in DMSO), and 2 µL of a horseradish peroxidase stock solution (5 mg/mL). The reaction was started by the addition of 20 µg of enzyme in 20 µl of phosphate buffer (50 mM, pH 7.4). One enzyme unit (U) was defined as the amount of enzyme that produced 1 µmol of hydrogen peroxide per min. The activity assays were performed in triplicate with the supernatant extract of vector PRSF-duet induced expression as control experiments.

Expression and purification of WT MAO-N and positive mutants

The WT and positive mutants were inoculated in 5 mL LB containing 50 µg/mL kanamycin and cultured overnight at 37 °C with shaking. The overnight cultures were scaled up to 800 mL TB containing 50 µg/mL kanamycin and induced by 0.5% lactose at 28°C for 20-22 hours. Then the cultures were harvested by centrifugation at 6,000 × g and resuspended in a PBS buffer (20 mM, pH 7.4) containing 500 mM NaCl, 20 mM imidazole. The cell pellets were disrupted by sonication and the cell debris was removed by centrifugation at 15,000 × g for 60 min. The soluble protein samples were loaded onto a nickel affinity column (GE Healthcare) and washed with 20~500 mM imidazole solution containing 500 mM NaCl and 20 mM PBS buffer (pH 7.4). The purified proteins were desalted and concentrated with centrifugation filtration devices. The protein concentrations were determined by Bradford method.

Determination of kinetic parameters

The kinetic parameters were obtained by measuring the initial velocities of the enzymatic reaction and curve-fitting according to the Michaelis-Menten equation. The activity assay was performed in a mixture containing a varying concentration of **1** (0.25-10 mM) and **2** (0.5-10 mM). All experiments were conducted in triplicate.

Preparative scale deracemization of substrates using recombinant cells of MAO-N mutants

Deracemization of substrate **1, 2, 4** and **5** were carried out as follows: Cell pellet from *E. coli* cultures (5 g) containing mutant LG-I-D11 or LG-J-B4 was resuspended in 98 mL of phosphate buffer (100 mM, pH 7.4). Substrate **1**(147 mg, 1.0 mmol) or **2** (104 mg, 0.5 mmol) or **4** (161 mg, 1mmol) or **5** (175 mg, 1mmol) in 2 mL of DMSO and borane–ammonia complex (124 mg, 4 mmol) were added and mixed. The mixture was shaken at 200 rpm and 30°C on an orbital shaker and the reaction was monitored by chiral HPLC analysis. When deracemization was finished, the pH of the reaction mixture was carefully adjusted to 11 with 5 M NaOH solution. The suspension was extracted three times with 100 mL of ethyl acetate and the phase separation was facilitated by centrifugation (6000 g, 15 min). The combined organic layer

was dried over anhydrous sodium sulfate and filtered. Removal of the solvent and purification by preparative thin layer chromatography gave the product.

Deracemization of **1** (147 mg) gave 108 mg product (73% isolated yield), which was identified as S isomer by comparison of the retention time on a HPLC with that of the authentic sample. The ee value (>99%) was determined by chiral HPLC analysis performed on an Agilent 1200 using Chiracel AD-H column (4.6 mm × 250 mm, DAICEL CHIRAL TECHNOLOGIES CO.LTD). A mixture of hexane, ethanol and hexane containing 0.5% diethylamine (88:2:10) was used as eluent at 1.0 mL/min of flow rate and the column temperature was controlled at 30°C. [1] The retention times for (S)- and (R)-**1** were 10.879 and 13.751 min, respectively. $[\alpha]^{25}_{D}$ = -57.4° (c = 0.6, EtOH); lit. $[\alpha]^{20}_{D}$ = -76.6° (c = 0.1, EtOH). [2] ^{1}H NMR (400 MHz, $CDCl_3$): δ 7.05 - 7.20 (m, 4 H), 4.17 (q, *J*=6.6 Hz, 1 H), 3.30 (dt, *J*=12.4, 5.3 Hz, 1 H), 3.06 (ddd, *J*=12.7, 8.3, 4.9 Hz, 1 H), 2.86 - 2.97 (m, 1 H), 2.75 - 2.82 (m, 1 H), 1.50 (d, *J*=6.8 Hz, 3 H). ^{13}CNMR (100 MHz, $CDCl_3$): δ 139.57, 134.30, 129.19, 126.19, 126.04, 125.91, 51.46, 41.39, 29.44, 22.33.

Deracemization of **2** (104 mg) gave 90 mg product (86% isolated yield), which was identified as S isomer by comparison of the retention time on a HPLC with that of the authentic sample. The ee value (93.4%) was determined by chiral HPLC analysis performed on an Agilent 1200 using Chiracel OD-H column (4.6 mm × 250 mm, DAICEL CHIRAL TECHNOLOGIES CO.LTD). A mixture of hexane and isopropanol (97:3) was used as eluent at 1.0 mL/min of flow rate and the column temperature was controlled at 40°C. [3] The retention times for (S)- and (R)-**2** were 8.597 and 13.172 min, respectively. $[\alpha]^{25}_{D}$ = +47.7° (c = 1.0, CH_2Cl_2); lit. $[\alpha]^{20}_{D}$ = +33.9° (c = 1.0, CH_2Cl_2). [3] ^{1}H NMR (400 MHz, $CDCl_3$): δ7.22 - 7.35 (m, 5 H), 7.14 (d, *J*=3.9 Hz, 2 H), 6.99 - 7.07 (m, 1 H), 6.75 (d, *J*=7.8 Hz, 1 H), 5.12 (s, 1 H), 3.22 - 3.31 (m, 1 H), 2.98 - 3.13 (m, 3 H), 2.79 - 2.89 (m, 1 H). ^{13}CNMR (100 MHz, $CDCl_3$): δ 144.49, 137.92, 135.30, 129.03, 128.44, 128.14, 127.47, 126.34, 125.70, 61.84, 41.97, 29.57.

Deracemization of **4** (161 mg) gave 129 mg product (80% isolated yield), which was identified as S isomer by comparison of the retention time on a HPLC with that of the authentic sample. The ee value (>99%) was determined by chiral HPLC analysis performed on an Agilent 1200 using Chiracel OD-H column (4.6 mm × 250 mm, DAICEL CHIRAL TECHNOLOGIES CO.LTD). A mixture of hexane/isopropanol/diethylamine (99:1:0.1) was used as eluent at 0.5 mL/min of flow rate and the column temperature was controlled at 40°C. [4] The retention times for (S)- and (R)-**4** were 18.465 and 21.592 min, respectively. $[\alpha]^{25}_{D}$ = -57.4° (c = 1.0, EtOH); lit. $[\alpha]^{20}_{D}$ = -71.1° (c = 0.8, EtOH). [5] ^{1}H NMR (400 MHz, $CDCl_3$): δ 7.03 - 7.18 (m, 4 H), 3.98 (dd, *J*=8.4, 3.5 Hz, 1 H), 3.90 (br. s., 1 H), 3.24 - 3.34 (m, 1 H), 3.03 (ddd, *J*=12.5, 7.8, 4.9 Hz, 1 H), 2.73 - 2.95 (m, 2 H), 1.95 (dqd, *J*=14.4, 7.3, 7.3, 7.3, 4.0 Hz, 1 H), 1.81 (dquin, *J*=14.9, 7.5, 7.5, 7.5, 7.5 Hz, 1 H), 1.03 (t, *J*=7.5 Hz, 3 H). ^{13}C NMR (100 MHz, $CDCl_3$): δ 138.20, 134.68, 129.20, 126.20, 126.16, 125.96, 56.92, 40.82, 29.28, 28.68, 10.46.

Deracemization of **5** (175 mg) gave 142 mg product (81% isolated yield), which was identified as S isomer by comparison of the retention time on a HPLC with that of the authentic sample. The ee value (>99%) was determined by chiral HPLC analysis performed on an Agilent 1200 using Chiracel OD-H column (4.6 mm × 250 mm, DAICEL CHIRAL TECHNOLOGIES CO.LTD). A mixture of hexane/isopropanol/*N*,*N*-diethylamine (99:1:0.1) was used as eluent at 0.5 mL/min of flow rate and the column temperature was controlled at 40°C. [4] The retention times for (S)- and (R)-5 were 12.111 and 12.969 min, respectively. $[\alpha]^{25}_{D}$ = -112.8° (c = 0.5, EtOH). Literature values for ***(R)*-5**: $[\alpha]^{20}_{D}$ = 113.8 ° (c = 0.5, $CHCl_3$). [6] ^{1}H NMR (400 MHz, $CDCl_3$): δ 7.05 - 7.17 (m, 4 H), 4.00 (d, *J*=3.4 Hz, 1 H), 3.30 - 3.39 (m, 1 H), 3.25 (br. s., 1 H), 2.84 - 2.99 (m, 2 H), 2.64 - 2.74 (m, 1 H), 2.30 - 2.41 (m, 1 H), 1.13 (d, *J*=6.8 Hz, 3 H), 0.77 (d, *J*=6.8 Hz, 3 H). ^{13}C NMR (100 MHz, $CDCl_3$): δ 137.93, 135.87, 129.09, 126.00, 125.90, 125.83, 60.90, 42.29, 32.34, 29.90, 20.20, 15.83.

General procedure for the deracemization

Deracemization of substrate 6, 7, 8, 9, 10, 11 and 12 were carried out as follows: Cell pellet from E. coli cultures (25 mg) containing mutant LG-I-D11 or LG-J-B4 was resuspended in 0.5 mL of phosphate buffer (100 mM, pH 7.4) containing borane–ammonia complex (80 mmol/L). Substrate in DMSO (5 µL, 1 mol/L) were added and mixed. The mixture was shaken at 200 rpm and 30°C on an orbital shaker for 24 h. HPLC samples were prepared as follows: aqueous NaOH-solution (50 µL, 5 M) was added to the reaction mixture in the Eppendorf tube, followed by 0.8 mL of methyl tert-butyl ether. After vigorous mixing,

the sample was centrifuged at 13000 x g for 1 minute. The organic phase was separated, dried over sodium sulfate and analyzed by chiral HPLC.

The configuration of 6, 7, 8, 9, 10 and 12 was determinate by comparison with literature HPLC retention time. The configuration of 11 was assigned by analogy with the other compounds.

Modelling and molecular dynamics simulation

The 3D structural models of WT MAO-N ,LG-F-B6, LG-J-B4 and LG-I-D11 were constructed based on X-ray crystal structures of the mutation *Aspergillus niger* MAO-N (PDB ID: 2VVL and 2VVM) using Schrodinger2015-3 [7] Energy minimization of the constructed model was done using AMBER16 [8]. Active site pocket volumes were calculated in Accelrys Discovery Studio 4.1 by searching for cavities. AMBER16 was used to carry out molecular dynamics simulation of final model using ff14SB.redq force field. The MD trajectories were further analyzed, along with the binding energy, to identify relevant binding poses and structures of entrance and exit channels that were sampled in the simulation. All atom molecular dynamics simulations have been performed using AMBER16 molecular dynamics package [8]. The bonded and non-bonded description of the interactions between the various atoms has been described using the AMBER16 force fields which include the ff14SB.redq force field parameters. The ANTECHAMBER module and GAFF2 with AM1-BCC charges[9] are used to obtain force field parameters for ligands. Initially, we perform a series of energy minimization steps to eliminate any bad contacts in the initially built structures. During the minimization, protein and FAD are restrained with harmonic force constants 200 kcal/mol. The minimization step involves 2500 steps steepest descent followed by 2500 steps of conjugate gradient method. After the energy minimization, the system is slowly heated up to 300 K in 100 ps MD using 1 fs integration time step, while restraining the solute with 20 kcal/mol harmonic force constant. After this, we perform 200 ps NPT equilibration of the structures with no harmonic restraints. Finally, 50 ns NPT production simulations are performed at 300 K and 1 atm pressure with 2 fs integration time step. We have implemented periodic boundary condition across the system using a TIP3P water box [10]. We use Particle Mesh Ewald (PME) techniques integrated with AMBER package to account for the long range part of the electrostatic interactions [11]. During the dynamics, all the bonds involving hydrogen are restrained using the SHAKE algorithm [12]. Langevin thermostat with collision frequency of 1 ps-1 is used to maintain the constant temperature while the pressure is controlled by anisotropic Monte-Carlo barostat [13]. The accelerated GPU version of PMEMD [14] was performed on Nvidia K40 series cards. We have employed CPPTRAJ [15] functionality of AMBERTOOLs [8] to perform various analyses on the equilibrium MD simulation trajectories. The images and graphics of the structures shown here were generated using the software packages VMD [16], Grace (version 5.1.25) [17] and PyMOL [18].

Supplementary Schemes，Figures and Tables

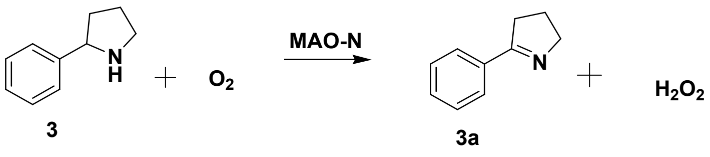

Scheme S1. Oxidation of amine 3 catalyzed by the monoamine oxidase MAO-N.

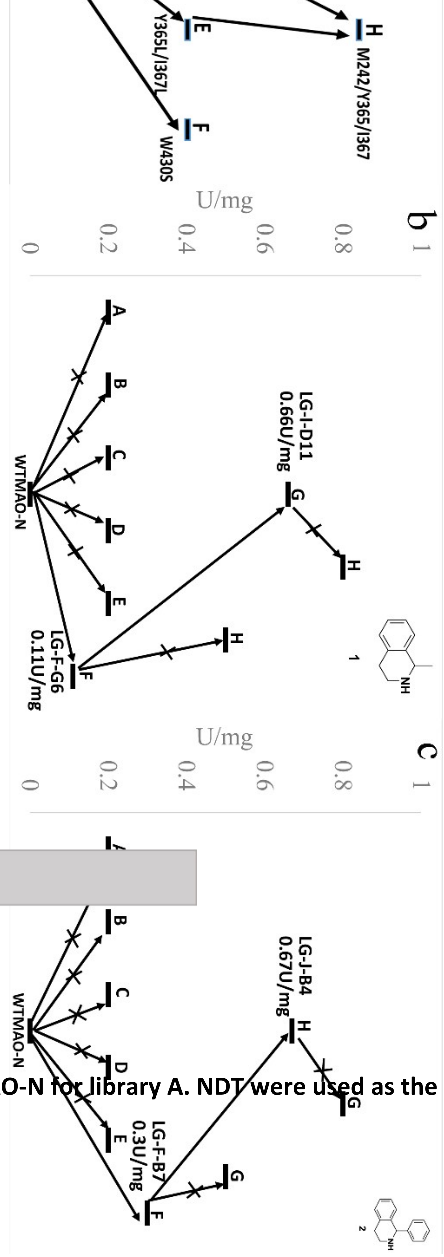

Figure S1. The directed evolution strategy of MAO-N toward amines **1** and **2**. a: Regrouping the functional residues recognized from the screening result of Libraries A, B, C, D, E and F for amine **3** into sites G and H. b: The ISM result of MAO-N for amine **1**. c: The ISM result of MAO-N for amine **2**.

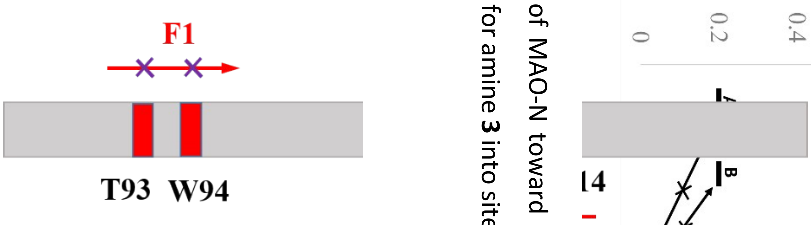

Figure S2. Primer design and libr[illegible] MAO-N for library A. NDT were used as the building blocks.

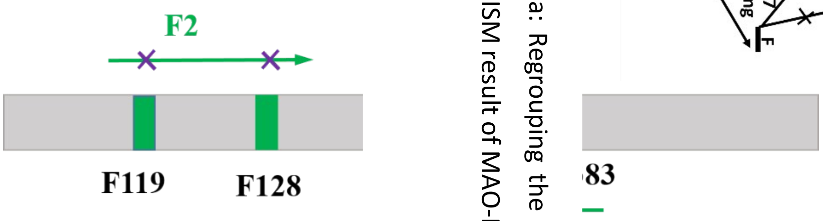

Figure S3. Primer design and libr[illegible] MAO-N for library B. NDT were used as the building blocks.

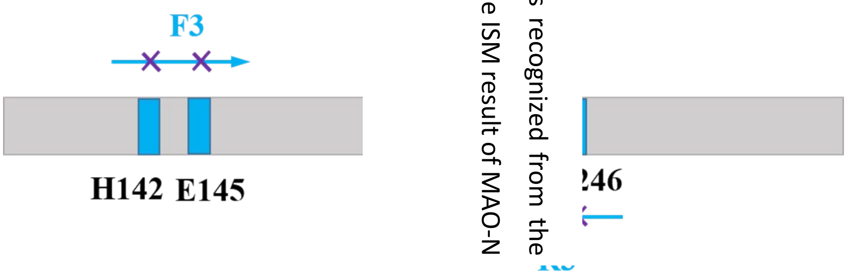

Figure S4. Primer design and library creation of WT MAO-N for library C. NDT were used as the building blocks.

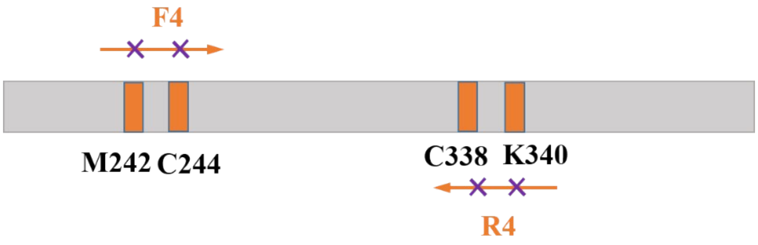

Figure S5. Primer design and library creation of WT MAO-N for library D. NDT were used as the building blocks.

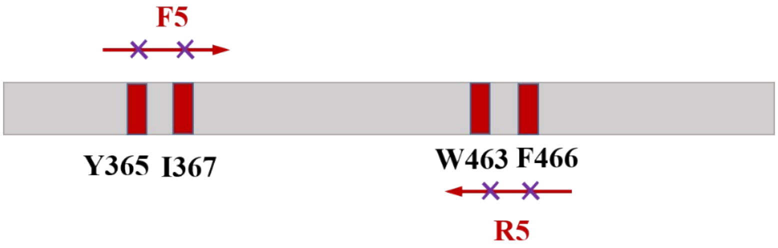

Figure S6. Primer design and library creation of WT MAO-N for library E. NDT were used as the building blocks.

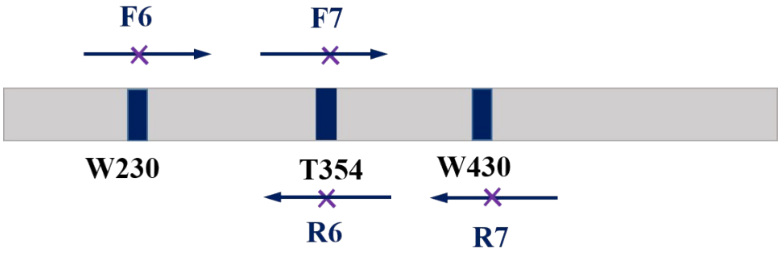

Figure S7. Primer design and library creation of WT MAO-N for library F. NDT were used as the building blocks.

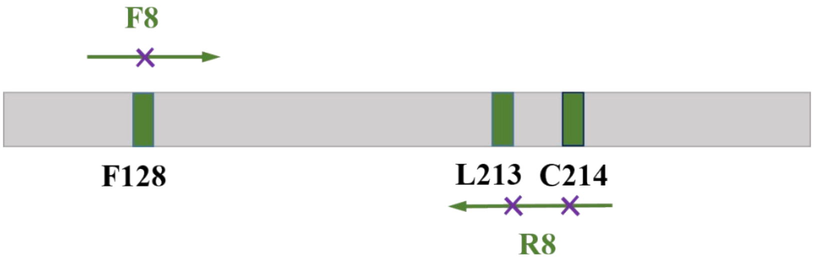

Figure S8. Primer design and library creation of WT MAO-N for library G. NDT were used as the building blocks.

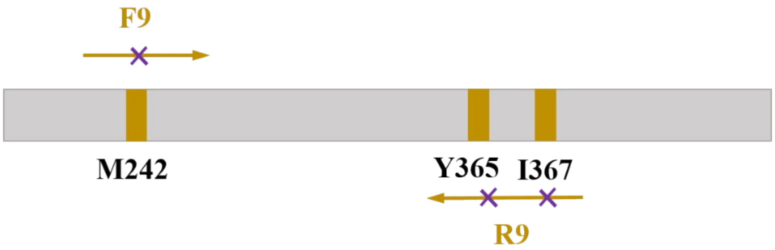

Figure S9. Primer design and library creation of WT MAO-N for library H. NDT were used as the building blocks.

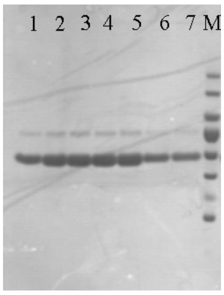

Figure S10. SDS-page of purified WT MAO-N and relative mutants. M: Marker, 1: WT MAON, 2: LG-F-B7, 3: LG-J-B4, 4: LG-F-G6, 5: LG-I-D11, 6: LG-F-B6 and 7: LG-F-B5.

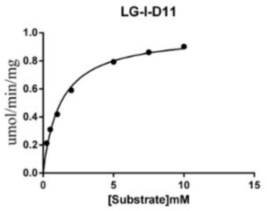

Michaelis-Menten	
Best-fit values	
Vmax	1.123
Km	1.1234
Std. Error	
Vmax	0.0256
Km	0.091

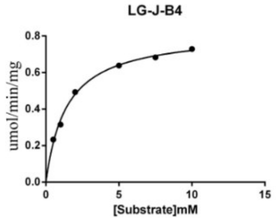

Michaelis-Menten	
Best-fit values	
Vmax	0.8233
Km	1.251
Std. Error	
Vmax	0.01026
Km	0.06252

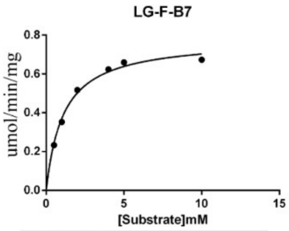

Michaelis-Menten	
Best-fit values	
Vmax	0.684
Km	1.421
Std. Error	
Vmax	0.013
Km	0.073

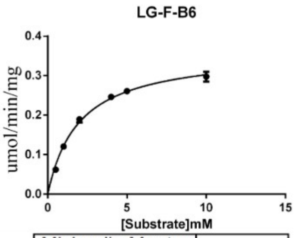

Michaelis-Menten	
Best-fit values	
Vmax	0.3617
Km	1.965
Std. Error	
Vmax	0.007876
Km	0.1239

Figure. S11 Kinetic analysis of MAON mutants (LG-I-D11 for substrate 1, LG-J-B4, LG-F-B7 and LG-F-B6 for substrate 2). Reactions were carried out in the presence of varying concentrations of 1 (0.25-10mM), and 2 (0.5-10mM). The Km and Vmax were determined from non-linear regression plots. Results are means ± SEM of triplicate experiments.

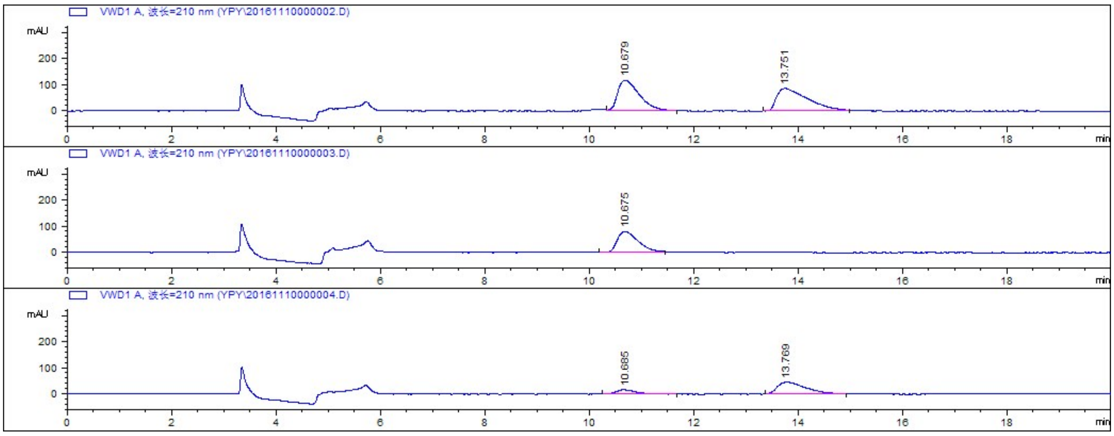

Figure S12. HPLC spectra of racemic 1, the product of variants LG-I-D11 ((*S*)-1) and MAO-N D5 (58.8% ee).

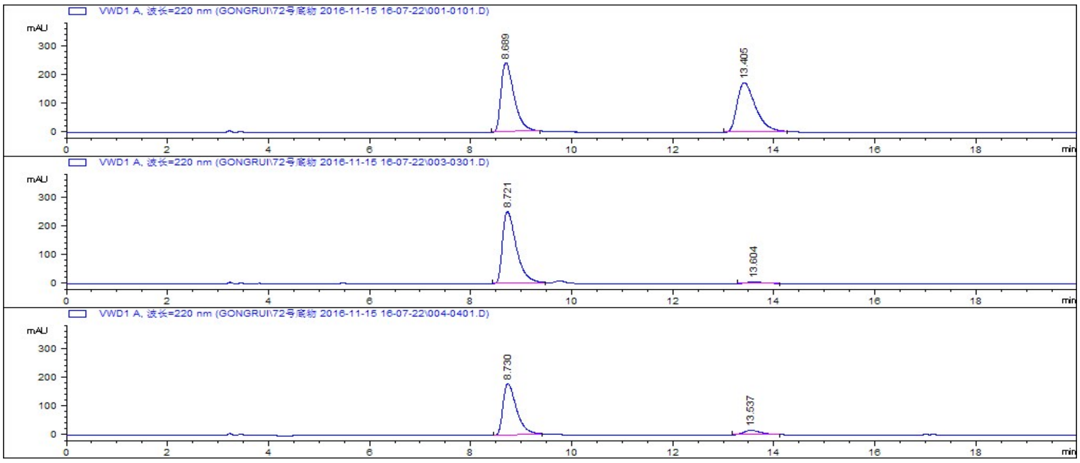

Figure S13. HPLC spectra of racemic 2 and the product of variants LG-J-B4 ((*S*)-2) and LG-I-D11 ((*S*)-2).

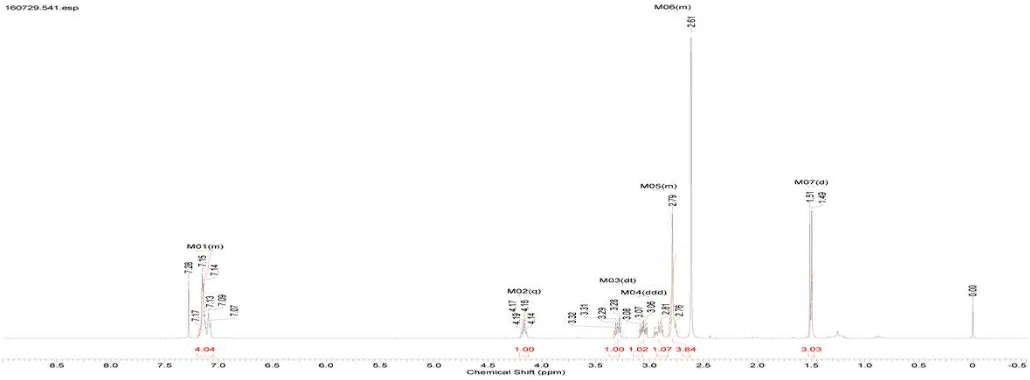

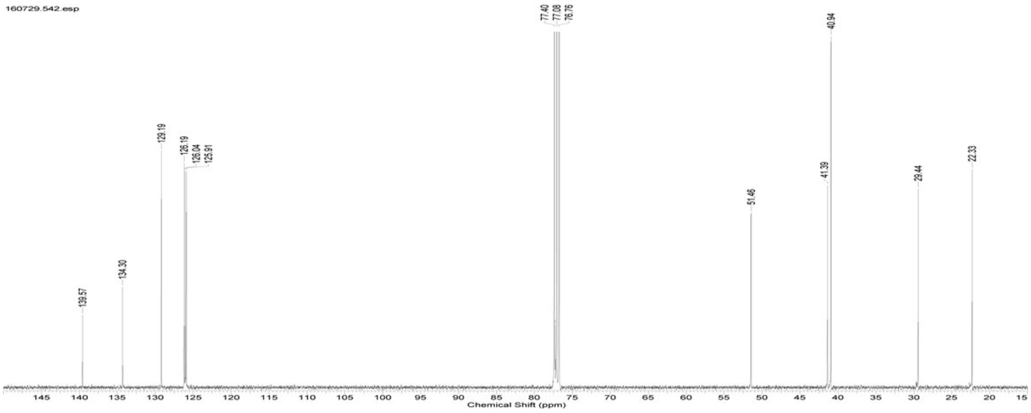

Figure S14. NMR spectra of the product (*S*)-1.

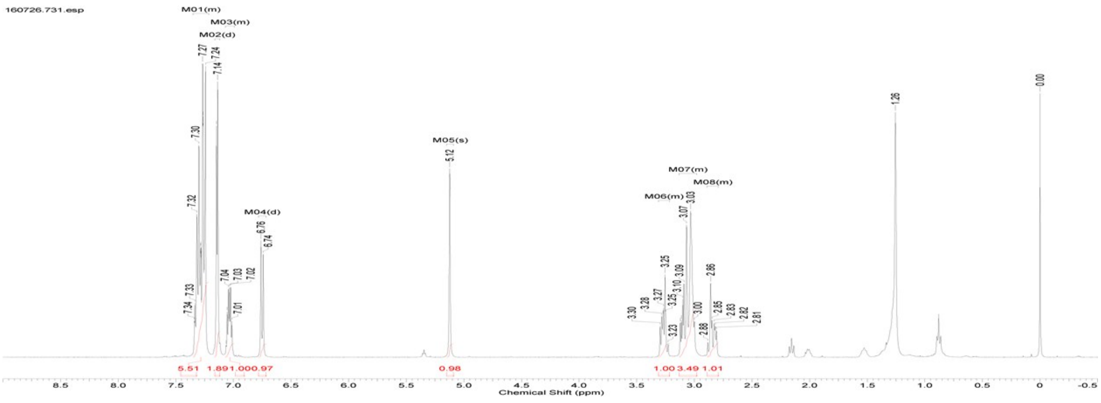

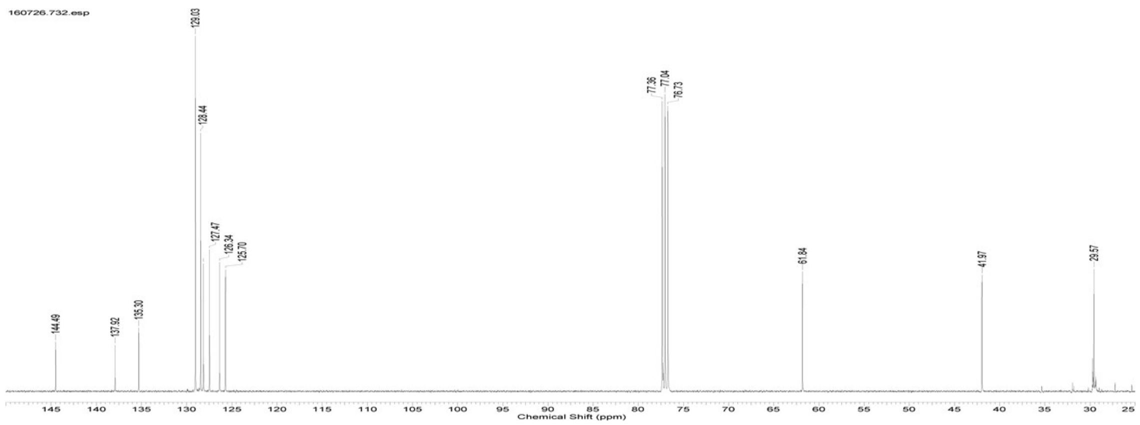

Figure S15. NMR spectra of the product (*S*)-2.

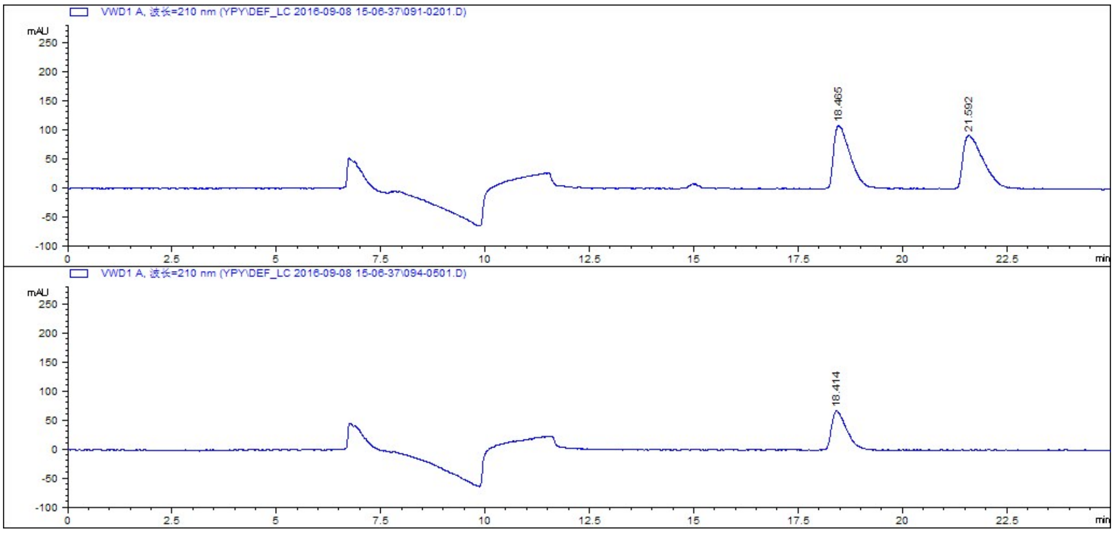

Figure S16. HPLC spectra of racemic 4 and the product (*S*)-4.

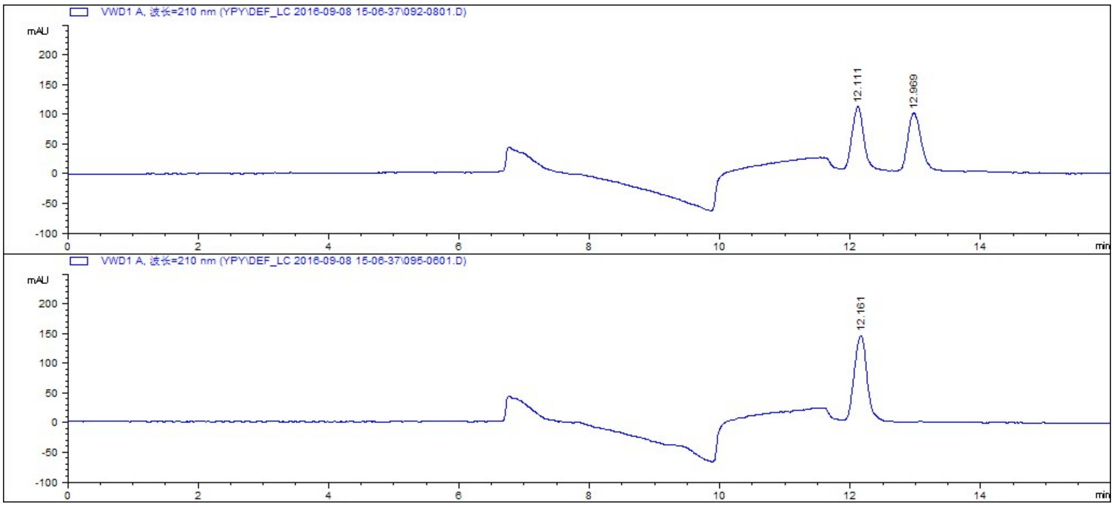

Figure S17. HPLC spectra of racemic 5 and the product (*S*)-5.

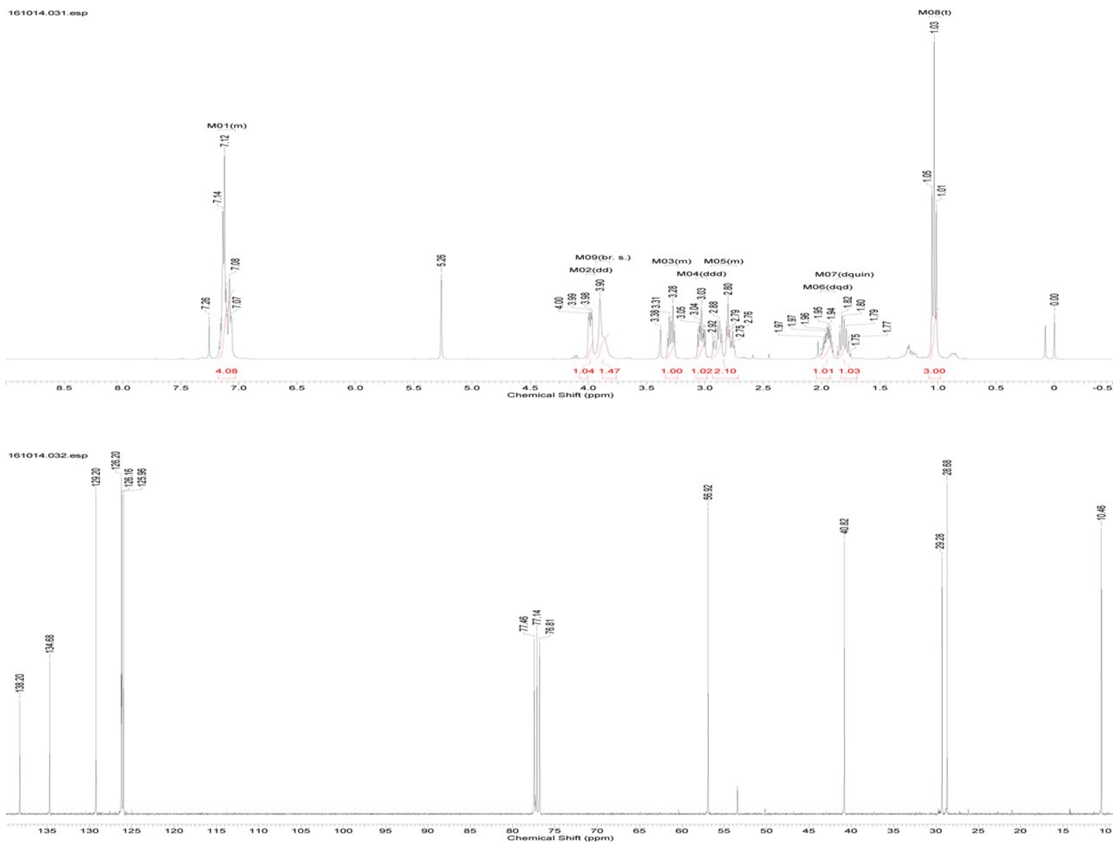

Figure S18. NMR spectra of the product (*S*)-4.

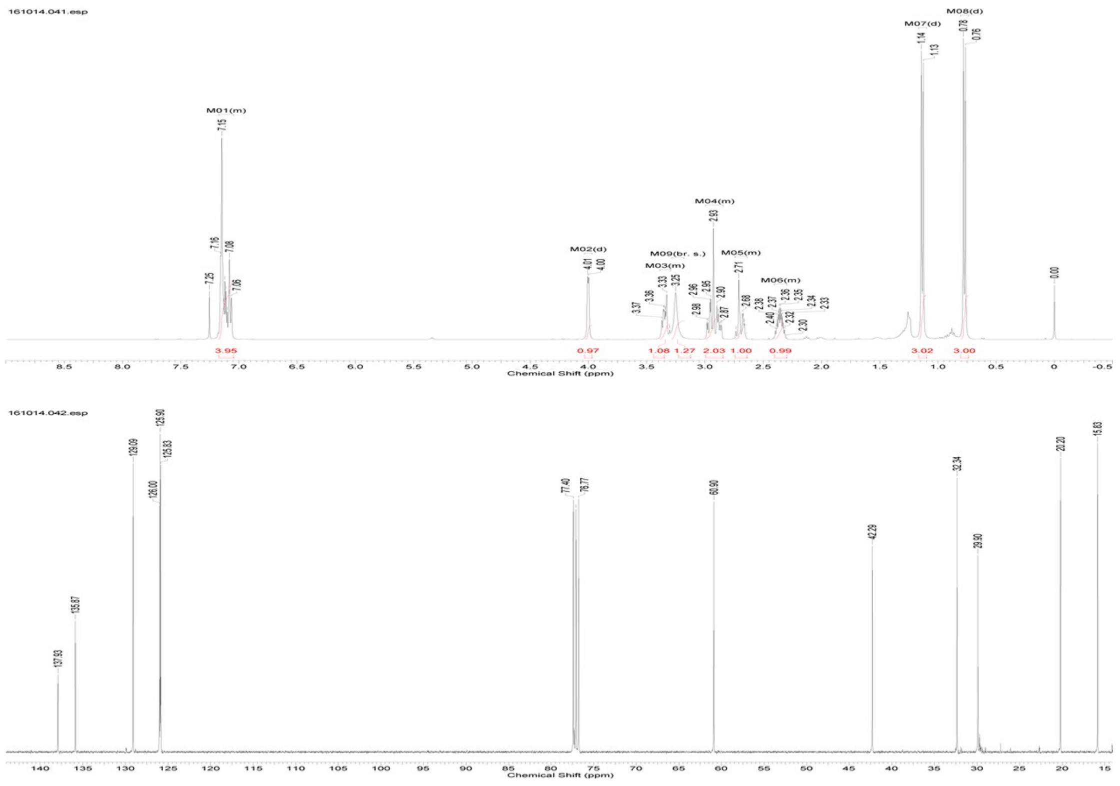

Figure S19. NMR spectra of the product (*S*)-5.

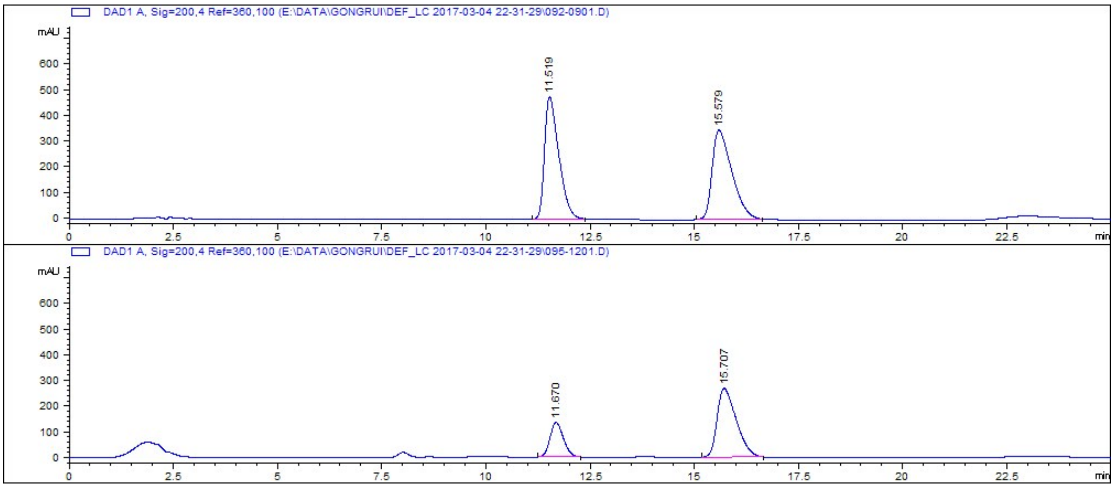

Figure S20. HPLC spectra of racemic 6 and the product of LG-J-B4

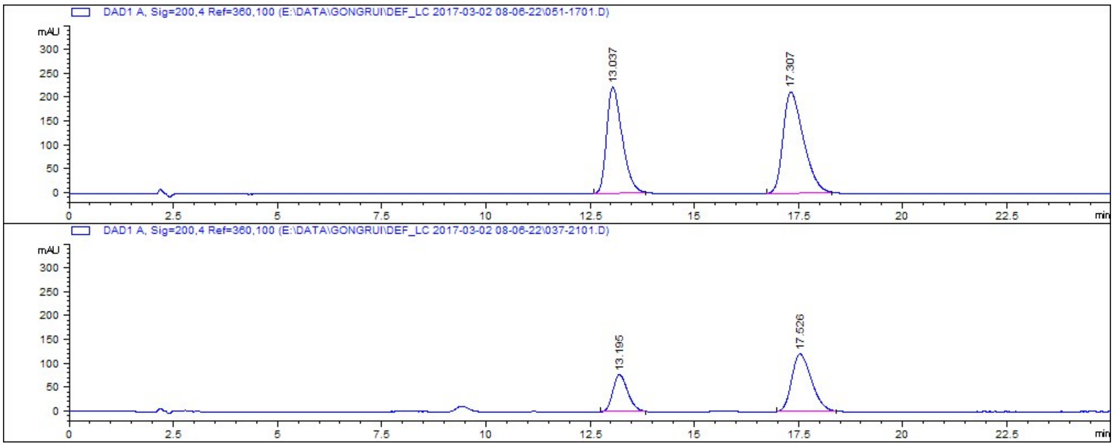

Figure S21. HPLC spectra of racemic 6 and the product of LG-I-D11

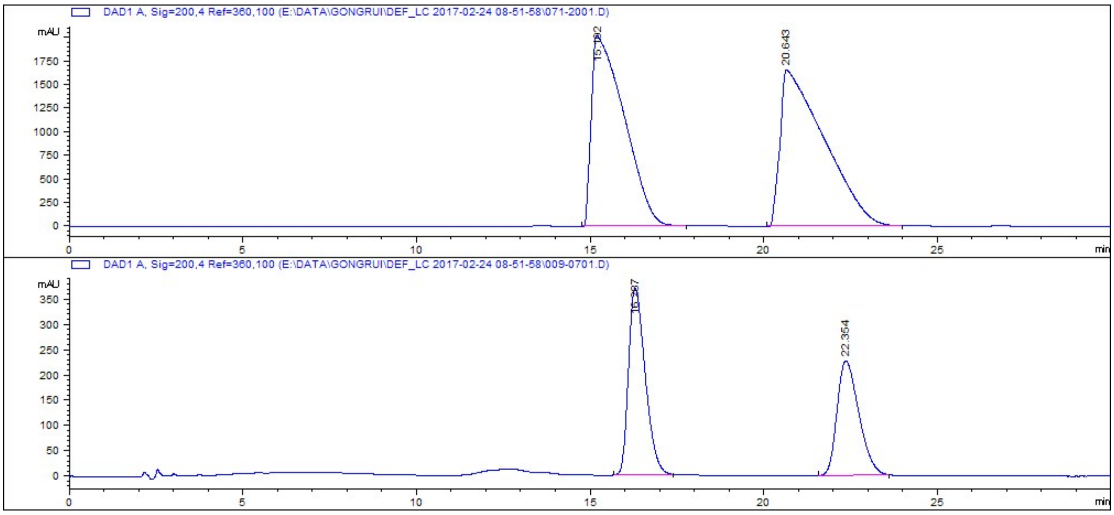

Figure S22. HPLC spectra of racemic 7 and the product of LG-J-B4

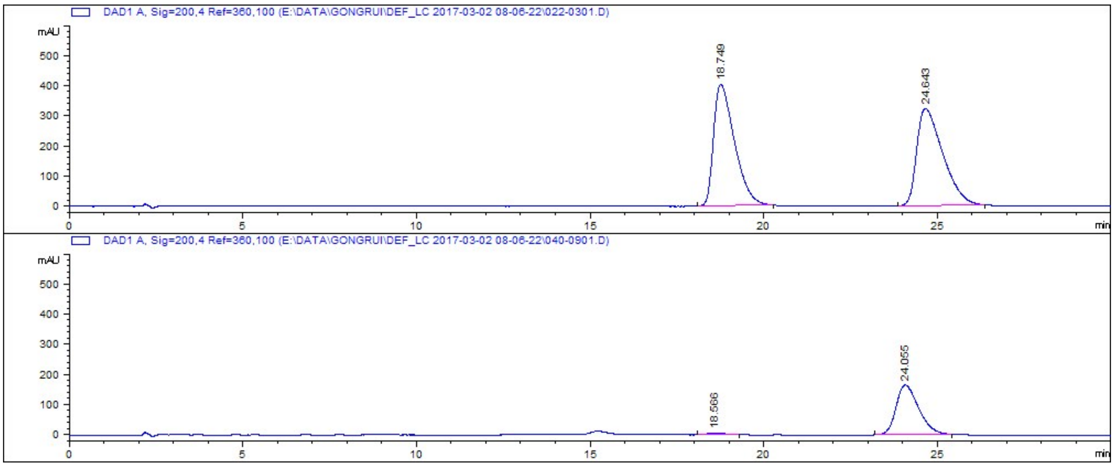

Figure S23. HPLC spectra of racemic 7 and the product of LG-I-D11

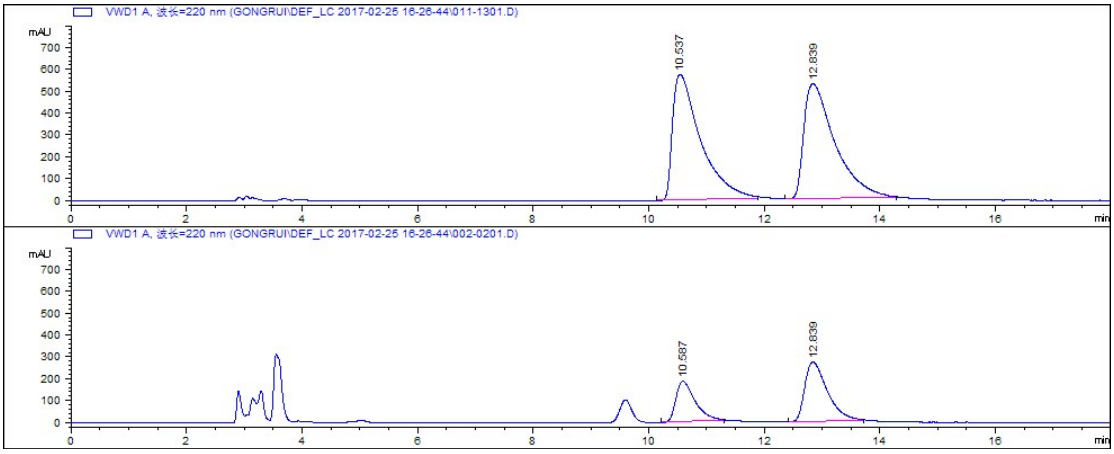

Figure S24. HPLC spectra of racemic 8 and the product of LG-J-B4

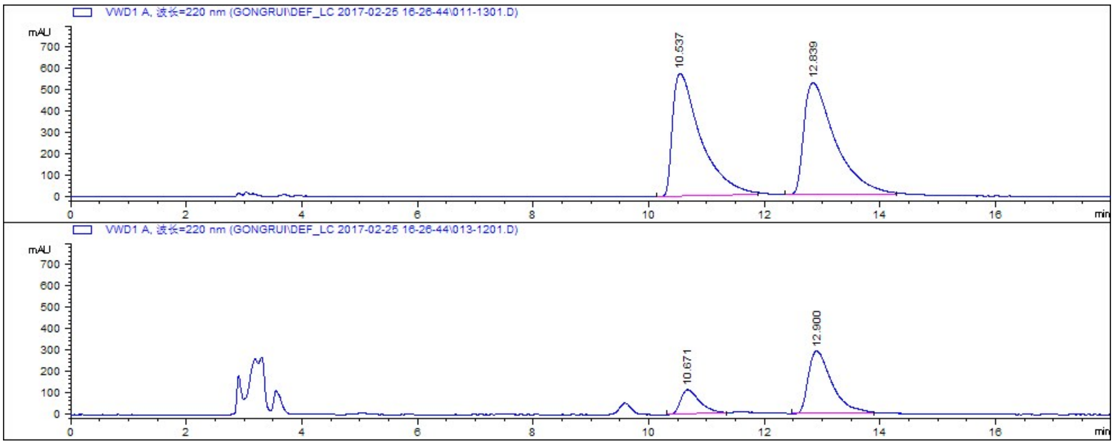

Figure S25. HPLC spectra of racemic 8 and the product of LG-I-D11

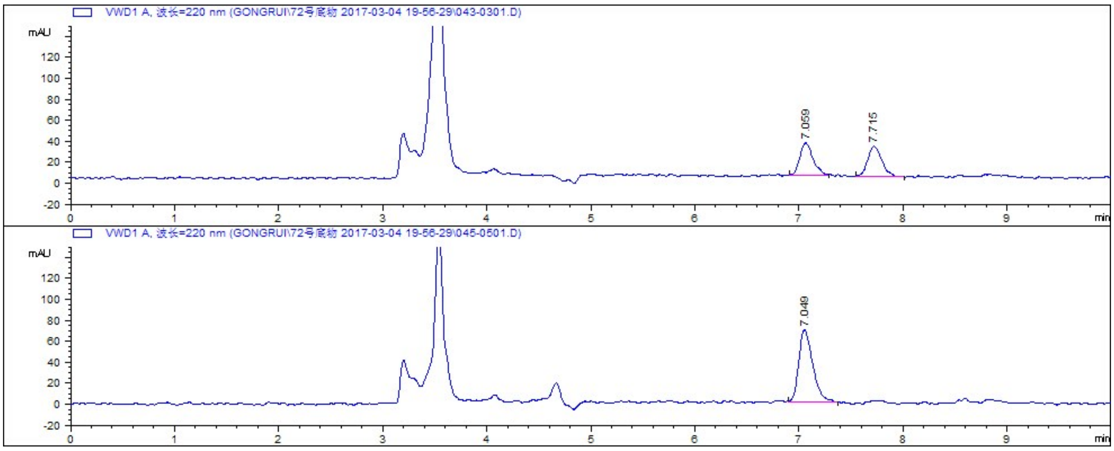

Figure S26. HPLC spectra of racemic 9 and the product of LG-I-D11

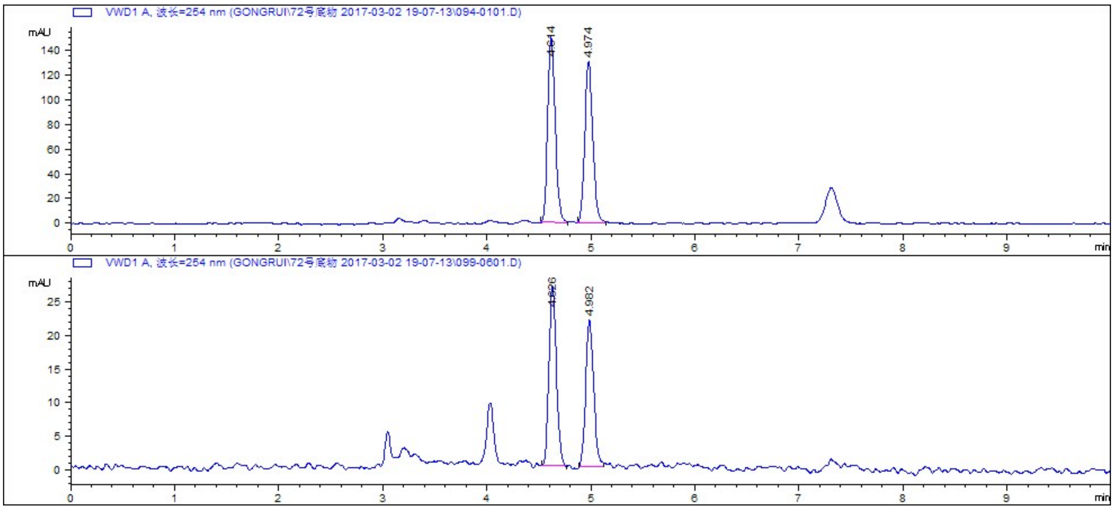

Figure S27. HPLC spectra of racemic 10 and the product of LG-J-B4

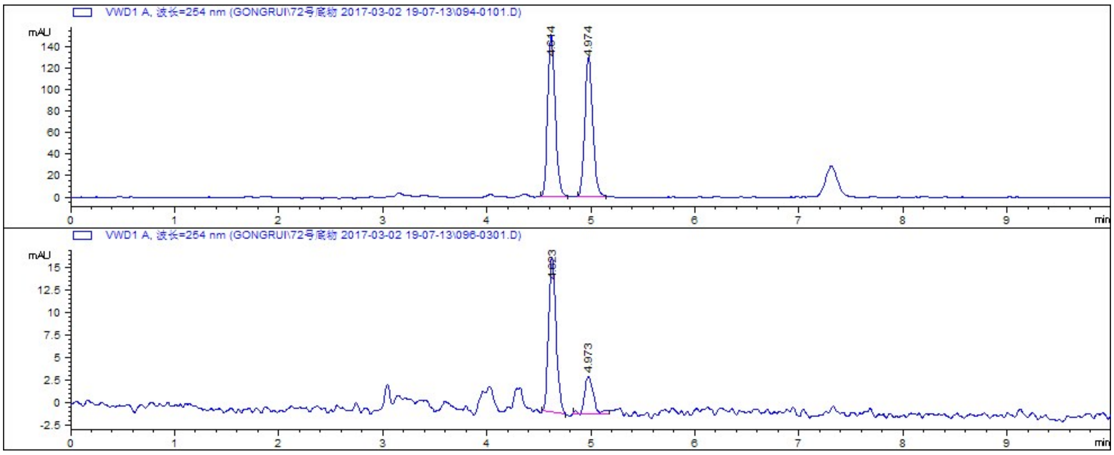

Figure S28. HPLC spectra of racemic 10 and the product of LG-I-D11

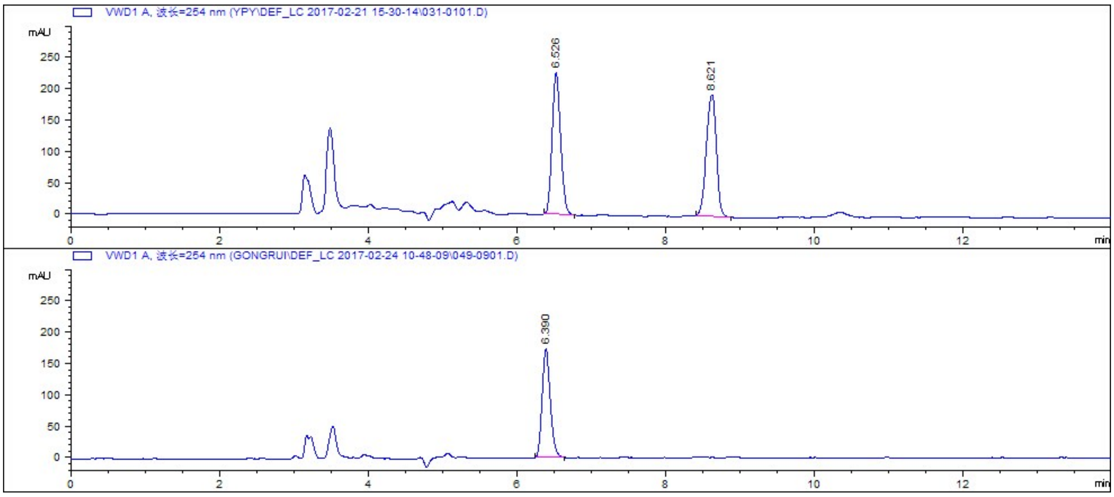

Figure S29. HPLC spectra of racemic 11 and the product of LG-J-B4

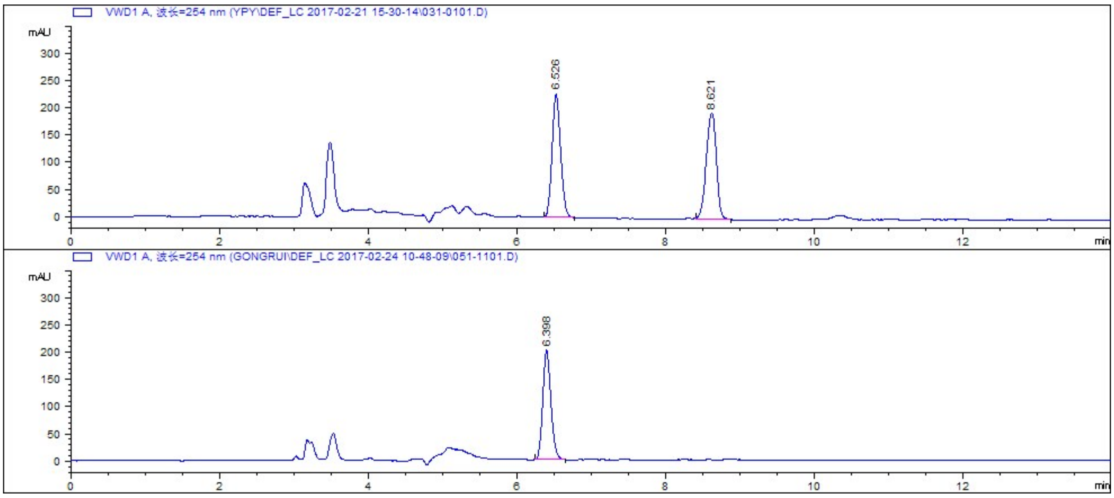

Figure S30. HPLC spectra of racemic 11 and the product of LG-I-D11

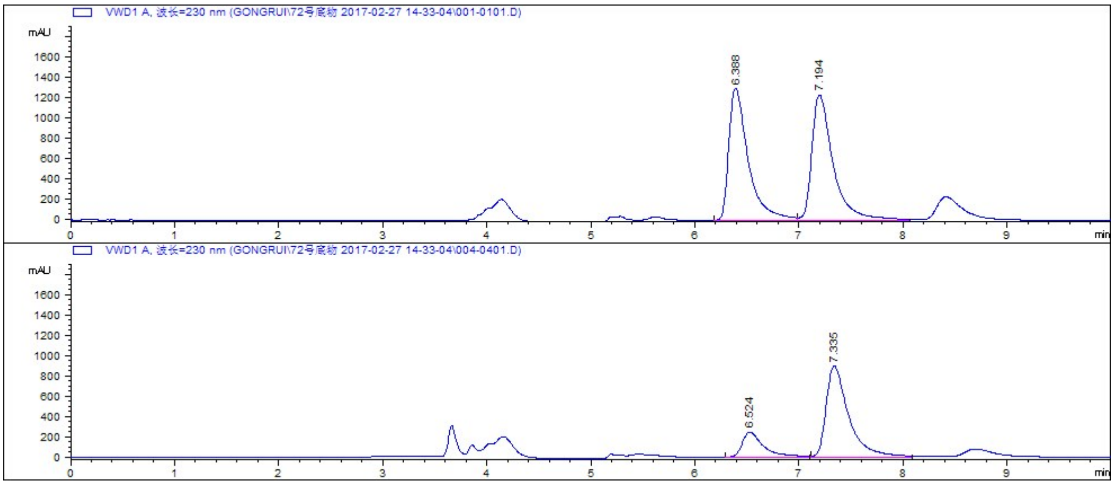

Figure S31. HPLC spectra of racemic 12 and the product of LG-I-D11

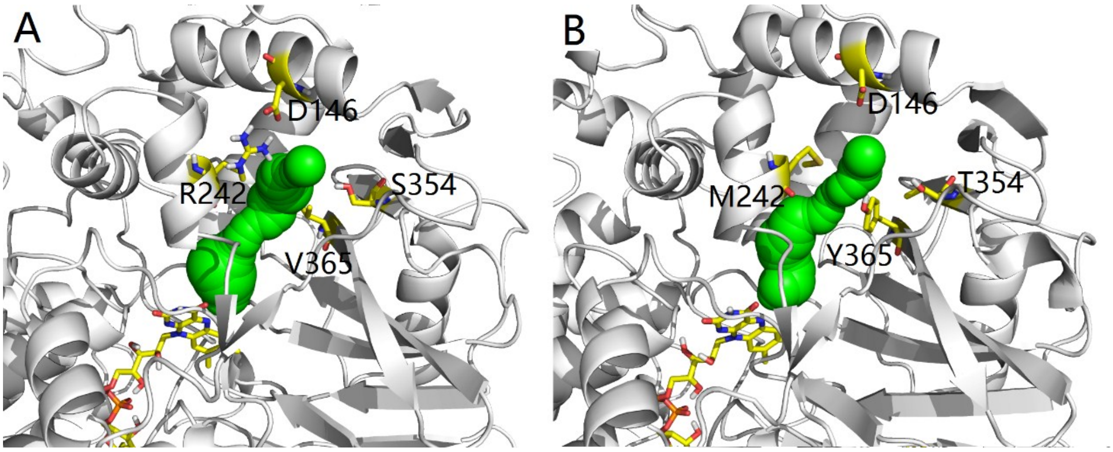

Figure S32. The green balls represent the tunnels of LG-F-B4 (A) and LG-J-B6 (B).

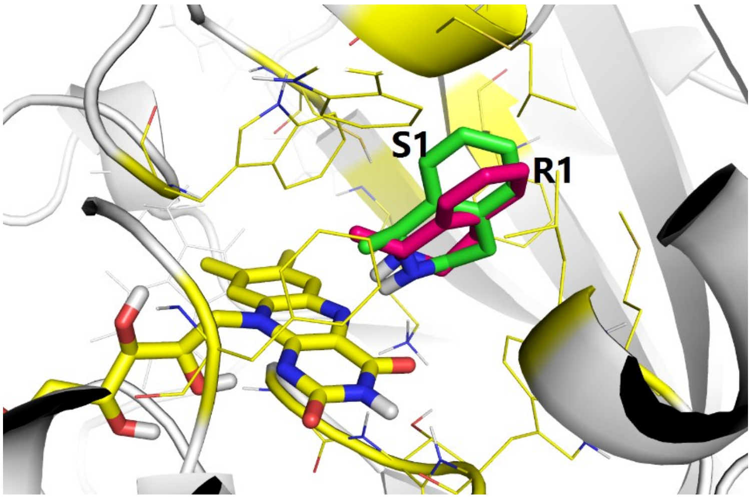

Figure S33. The superimposition of the binding models of the respective complexes (*R*)-1 and (*S*)-1 to LG-I-D11.

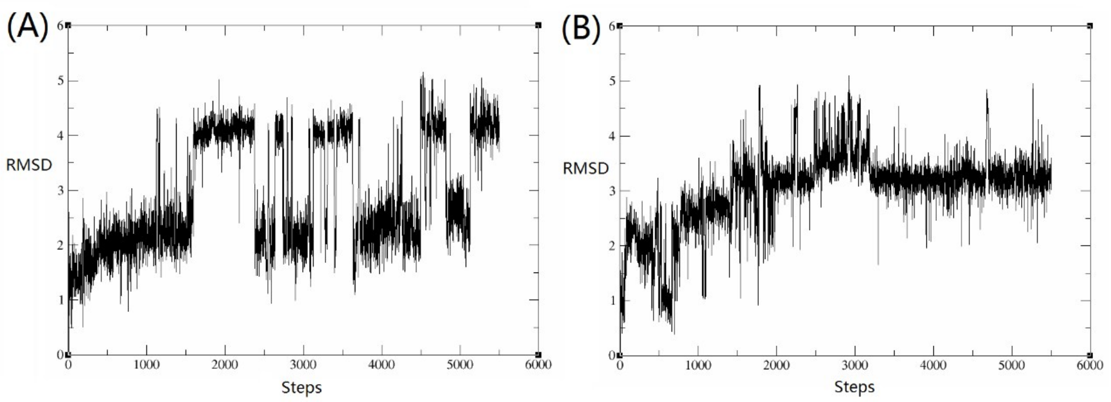

Figure S34. The rmsd compute of LG-I-D11-*R*1 (A) and LG-I-D11-*S*1 (B).

Table S1. Results of screening libraries of MAO-N for amine 3 using code NDT.

Library	Code	Mutation	Activity
template	WT		–
**A**	LG-A-A1	L213I/C214I	–
	LG-A-A2	L213V/C214L	+
	LG-A-A3	L213V/C214I	+
template	WT		–
**B**	LG-B-B8	F128H	+

template	WT		–
**C**	ND		
template	WT		–
**D**	LG-D-D1	M242L	++
	LG-D-D7	M242R	+
	LG-D-D8	M242G	+
template	WT		–
**E**	LG-E-E4	Y365L/I367L	+
	LG-E-E6	Y365L/I367H	–
template	WT		–
**F**	LG-F-E2	T354N/W430S	++
	LG-F-F10	W430S	+++
	LG-F-G4	W230I/T354H	–
	LG-F-G6	W230R/W430C	++

ND: not detected; –: Activity is similar or lower than wtMAO-N; +, ++, +++, ++++: Activity is higher than wtMAO-N and increase gradually.

Table S2. Results of screening libraries of MAO-N for amine 1 using code NDT.

Library	Code	Mutation	Activity
template	WT		ND
A	ND		
template	WT		ND
**B**	ND		
template	WT		ND

**C**	ND		
template	WT		ND
**D**	ND		
template	WT		ND
**E**	ND		
template	WT		ND
**F**	LG-F-A5	W430C	+
	LG-F-D12	W230L/W430G	+
	LG-F-F10	W430S	+
	LG-F-G4	W230I/T354H	+
	LG-F-G6	W230R/W430C	++
template	LG-F-G6	W230R/W430C	++
**F→G**	LG-I-D11	W230R/W430C/C214L	++++
template	LG-F-G6	W230R/W430C	++
**F→H**	ND		
template	LG-I-D11	W230R/W430C/C214L	++++
**F→G→H**	ND		

ND: not detected; –: Activity is similar or lower than wtMAO-N; +, ++, +++, ++++: Activity is higher than wtMAO-N and increase gradually.

Table S3. Results of screening libraries of MAO-N for amine 2 using code NDT.

Library	Code	Mutation	Activity
template	WT		ND
A	ND		

template	WT		ND
**B**	ND		
template	WT		ND
**C**	ND		
template	WT		ND
**D**	ND		
template	WT		ND
**E**	ND		
template	WT		ND
**F**	LG-F-B7	W230I/T354S/W430R	++
	LG-F-C7	W430G	+
	LG-F-D8	W230L/T354R/W430R	+
	LG-F-F8	W230I/T354S/W430N	+
	LG-F-G4	W230I/T354H	+
	LG-F-G6	W230R/W430C	+
template	LG-F-B7	W230I/T354S/W430R	++
**F→G**	ND		
template	LG-F-B7	W230I/T354S/W430R	++
**F→H**	LG-J-B4	W230I/T354S/W430R/M242R/Y365V	++++
template	LG-J-B4	W230I/T354S/W430R/M242R/Y365V	++++
**F→H→G**	ND		

ND: not detected; –: Activity is similar or lower than wtMAO-N; +, ++, +++, ++++: Activity is higher than wtMAO-N and increase gradually.

Table S4. List of primers for MAO-N libraries A-H.

Library	Primers	Sequence (5' to 3')
A	F1	AGATGGGTGGC**NDTNDT**GTTCATTGGCATCAAT
	R1	GGGTGCCACCGCT**AHNAHN**TAAAATGAAAGCCT
B	F2	CGCTGTCTCCATCA**NDT**AACTTTTCACGTGGCGTGAATCAC**NDT**CAACTGCGTACCAATC
	R2	TTCGCGTCTGT**AHNAHN**GCACACCAGATGCGTA
C	F3	TACGTACATGACG**NDT**GAAGCG**NDT**GATGAACTGCTGCGTAGCG
	R3	AAATTTATAGGA**AHNAHN**ACAATCCATACAGCCC
D	F4	GTATCAGGGCTGT**NDT**GAT**NDT**TTAATCTCCTATAAATTTA
	R4	CTTCCGCATGCAC**AHN**GGT**AHN**CATGTTAACATGGCCTG
E	F5	TTCAACAAATTATGC**NDT**GCT**NDT**GGCGATGGCACGACCCCAG
	R5	AGCACCATCGAT**AHN**ACTACG**AHN**ACCTAAGGCCCAATCG
F	F6	TGAATTTCTGCAT**NDT**TGGGCAATGTCGGGTTA
	R6	TGAACGGATAGGCGATACC**AHN**CCATGAACGCATATCCT
	F7	AGGATATGCGTTCATGG**NDT**GGTATCGCCTATCCGTTCA
	R7	CTGGGCGGCTGAAGAA**AHN**CGCGCCTTTGGCAAATT
G	F8	GCGTGAATCAC**NDT**CAACTGCGTACCAA
	R8	GGGTGCCACCGCT**AHNAHN**TAAAATGAAAGCCT
H	F9	ATCAGGGCTGT**NDT**GATTGTTTAATCTC
	R9	TCGTGCCATCGCC**AHN**AGC**AHN**GCATAATTTGTTGAACG

Table S5 HPLC columns, conditions and retention times.

Compound	Column	Eluent	Flow(mL/min)	Retention time (min)	reference
rac-6	CHIRALPAK CR(+) (4.0mm×150mm)	$HClO_4$(pH3):MeOH (90:10)	0.5	13.0(*S*) 17.3(*R*)	[20]
rac-7	CHIRALPAK CR(+) (4.0mm×150mm)	$HClO_4$(pH3): MeOH (90:10)	0.5	18.7(*S*) 24.6(*R*)	[20]
rac-8	CHIRALCEL OD-H (4.6mm×250mm)	Hexane: iPrOH (90:10)	1.0	10.5(*S*) 12.8(*R*)	[3]
rac-9	CHIRALPAK IC (4.6mm×250mm)	Hexane:iPrOH: diethylamine (90:10: 0.1)	1.0	7.0(*S*) 7.7(*R*)	[21]
rac-10	CHIRALPAK IC (4.6mm×250mm)	Hexane:iPrOH: diethylamine (90:10: 0.1)	1.0	5.0(*S*) 4.6(*R*)	[22]
rac-11	CHIRALPAK IC (4.6mm×250mm)	Hexane:iPrOH: diethylamine (90:10: 0.1)	1.0	6.5(*S*) 8.6(*R*)	[21]
rac-12	CHIRALCEL OD-H (4.6mm×250mm)	Hexane:iPrOH (98:2)	0.8	7.2(*S*) 6.4(*R*)	[23]

Table S6: The MM-GBSA predicted binding free energy for LG-I-D11-R1 and LG-I-D11-S1.

	[1]ELE	[2]VDW	[3]INT	[4]GAS	[5]GBSUR	[6]GB	[7]GBSOL	[8]GBELE	[9]GBTOT
R1	-8.57	-26.36	0.49	-34.43	-2.17	12.91	10.74	4.34	-23.69
S1	-4.85	-24.18	0.57	-28.46	-2.01	13.78	11.76	8.92	-16.70

[1]ELE - non-bonded electrostatic energy + 1,4-electrostatic energy; [2]VDW – non-bonded van der Waals energy + 1,4-van der Waals energy; [3]INT – bond, angle, dihedral energies; [4]GAS – ELE + VDW + INT; [5]GBSUR –Free

energy for GB calculations; [6]GB – reaction field energy calculated by GB; [7]GBSOL – GBSUR + GB; [8]GBELE – GB + ELE; [9]GBTOT – GBSOL + GAS.

References

[1] J. M. Foulkes, K. J. Malone, V. S. Coker, N. J. Turner and J. R. Lloyd, *ACS Catal.*, 2011, **1**, 1589-1594.
[2] V. M. Dem'yanovich, I. N. Shishkina, *Chem. Heterocycl. Comp.,* 1996, **32**, 807-811.
[3] D. Ghislieri, A. P. Green, M. Pontini, S. C. Willies, I. Rowles, A. Frank, G. Grogan and N. J. Turner, *J. Am. Chem. Soc.*, 2013, **135**, 10863-10869.
[4] C. Q. Li, J. L. Xiao, *J. Am. Chem. Soc.,* 2008, **130**, 13208-13209.
[5] Z. Y. Ding, T. L. Wang, Y. M. He, F. Chen, H. F. Zhou, Q. H. Fan, Q. X. Guo, A. S. C. Chan, *Adv. Synth. Catal.,* 2013, **355**, 3727-3735.
[6] Y. Ji, L. Shi, M. W. Chen, G. S. Feng, Y. G. Zhou, *J. Am. Chem. Soc.,* 2015, **137**, 10496-10499.
[7] Schrödinger Suite 2015 Protein Preparation Wizard; Epik version 3.2, Schrödinger, LLC, New York, NY, 2015; Impact version 6.7, Schrödinger, LLC, New York, NY, 2015; Prime version 4.0, Schrödinger, LLC, New York, NY, 2015.
[8] D.A. Case, R.M. Betz, W. Botello-Smith, D.S. Cerutti, T.E. Cheatham, III, T.A. Darden, R.E. Duke, T.J. Giese, H. Gohlke, A.W. Goetz, N. Homeyer, S. Izadi, P. Janowski, J. Kaus, A. Kovalenko, T.S. Lee, S. LeGrand, P. Li, C. Lin, T. Luchko, R. Luo, B. Madej, D. Mermelstein, K.M. Merz, G. Monard, H. Nguyen, H.T. Nguyen, I. Omelyan, A. Onufriev, D.R. Roe, A. Roitberg, C. Sagui, C.L. Simmerling, J. Swails, R.C. Walker, J. Wang, R.M. Wolf, X. Wu, L. Xiao, D.M. York and P.A. Kollman, AMBER 2016, University of California, San Francisco.
[9] A. Jakalian, B. L. Bush, D. B. Jack and C. I. Bayly, *J. Comput. Chem.,* 2000, **21**, 132-146.
[10] W. L. Jorgensen, J. Chandrasekhar, J. D. Madura, R. W. Impey and M. L. Klein, *J. Chem. Phys.*, 1983, **79**, 926-935.
[11] D. A. Pearlman, D. A. Case, J. W. Caldwell, W. S. Ross, T. E. Cheatham, S. DeBolt, D. Ferguson, G. Seibel and P. Kollman, *Comput. Phys. Commun.*, 1995, **91**, 1-41.
[12] H. C. Andersen, *J. Chem. Phys.*, 1983, **52**, 24-34.
[13] K.-H. Chow and D. M. Ferguson, *Comput. Phys. Commun.*, 1995, **91**, 283-289.
[14] S. Le Grand, A. W. Götz and R. C. Walker, *Comput. Phys. Commun.*, 2013, **184**, 374-380.
[15] D. R. Roe and T. E. Cheatham III, *J. Chem. Theory Comput.*, 2013, **9**, 3084-3095.
[17] W. Humphrey, A. Dalke and K. Schulten, *J. Mol. Graph.*, 1996, **14**, 33-38.
[18] Grace, http://plasma-gate.weizmann.ac.il/Grace.
[19] DeLano, W.L. (2002) The Pymol Molecular Graphics System. DeLano Scientific, San Carlos, California, USA.
[20] J. Jiang, X. Chen, D. Zhang, Q. Wu and D. Zhu, *Appl. Microbiol. Biotechnol.*, 2015, **99**, 2613-2621.
[21] F. Leipold, S. Hussain, D. Ghislieri and N. J. Turner, *ChemCatChem*, 2013, **5**, 3505-3508.
[22] R. S. Heath, M. Pontini, S. Hussain and N. J. Turner, *ChemCatChem*, 2015, **8**, 117-120.
[23] P. C. Yan, J. H. Xie, G. H. Hou, L. X. Wang and Q. L. Zhou, *Adv. Synth. Catal.*, 2009, **351**, 3243-3250.
